# Construction of a new immune-related lncRNA model and prediction of treatment and survival prognosis of human colon cancer

**DOI:** 10.1186/s12957-022-02508-2

**Published:** 2022-03-06

**Authors:** Sicheng Liu, Xingyu Peng, Xun Wu, Fanqin Bu, Zhonglin Yu, Jinfeng Zhu, Chen Luo, Wenjun Zhang, Jiang Liu, Jun Huang

**Affiliations:** Department of Gastrointestinal Surgery, Second Affiliated Hospital of Nanchang University, Nanchang, 330006 Jiangxi China

**Keywords:** lncRNA, Colon cancer, Tumor-infiltrating immune cell, Immunotherapy

## Abstract

**Background:**

An increasing number of studies have shown that immune-related long noncoding RNAs (lncRNAs) do not require a unique expression level. This finding may help predict the survival and drug sensitivity of patients with colon cancer.

**Methods:**

We retrieved original transcriptome and clinical data from The Cancer Genome Atlas (TCGA), sorted the data, differentiated mRNAs and lncRNAs, and then downloaded immune-related genes. Coexpression analysis predicted immune-related lncRNAs (irlncRNAs) and univariate analysis identified differentially expressed irlncRNAs (DEirlncRNAs). We have also amended the lasso pending region. Next, we compared the areas under the curve (AUCs), counted the Akaike information standard (AIC) value of the 3-year receiver operating characteristic (ROC) curve, and determined the cutoff point to establish the best model to differentiate the high or low disease risk group of colon cancer patients.

**Results:**

We reevaluated the patients regarding the survival rate, clinicopathological features, tumor-infiltrating immune cells, immunosuppressive biomarkers, and chemosensitivity. A total of 155 irlncRNA pairs were confirmed, 31 of which were involved in the Cox regression model. After the colon cancer patients were regrouped according to the cutoff point, we could better distinguish the patients based on adverse survival outcomes, invasive clinicopathological features, the specific tumor immune cell infiltration status, high expression of immunosuppressive biomarkers, and low chemosensitivity.

**Conclusions:**

In this study, we established a characteristic model by pairing irlncRNAs to better predict the survival rate, chemotherapy efficacy, and prognostic value of patients with colon cancer.

**Supplementary Information:**

The online version contains supplementary material available at 10.1186/s12957-022-02508-2.

## Background

Colon cancer is a universal malignant tumor of the digestive tract. The incidence and death rates in China have increased in the last ten years [1]. Although surgery, chemotherapy, and targeted therapy have significantly improved the overall survival rate of patients, approximately 50% of colon cancer patients have distant metastasis, which is the most common cause of treatment failure [2]. With tumor metastasis, the 5-year survival rate is reduced to 8.1% [3]. Immunotherapy in the treatment of metastatic colorectal cancer has recently improved. Currently, the main diagnostic methods of colon cancer include biopsy, laboratory examination, and colonoscopy [4]. Immunotherapy (including antagonists of immune checkpoints, such as PD-1 and CTLA4) works best when combined with tumor vaccines or therapeutic agents that induce immune cell death [5]. For example, in some rodent models of colon cancer, OXP causes immunogenic cell death. To date, the immunogenicity of cell death induced by OXP is governed by the same rules as those induced by anthracyclines, involving CRT exposure, HMGB1 release, and the presence of functional TLR4 in the immune system [6].

LncRNAs constitute a class of noncoding RNAs (ncRNAs) greater than 200 nucleotides in length that play a vital role in cancer development [7]. LncRNAs are abundant, accounting for approximately 80% of the human transcriptome. They adjust 70% of human gene expression and do not act universally because they could affect DNA, RNA, and protein and show enhanced or inhibitory effects. Long noncoding RNAs (lncRNAs) play a key role in the carcinogenesis and progression of various human malignant tumors, including colon cancer [8]. The latest research findings show that lncRNA BC200 can be used as a new oncogene and therapeutic target for colon cancer [9]. A previous study found that lncRNA HOTAIR acts by cutting miRNA34a to promote the development of colon cancer [10]. LncRNA RAMS11 directly affects mCRC biology, including promoting an aggressive phenotype and being related to treatment response and resistance [11]. These studies indicate that the lncRNA expression level affects the development and survival of colon cancer.

Therefore, a credible risk evaluation model must be established to evaluate the survival prognosis of patients with colon cancer and optimize clinical decision-making. LncRNAs can help these signal models [12]. Identifying immune-related 9-lncRNA signaling can not only improve the ability of colon cancer patients to predict prognosis but also facilitate better clinical strategies and elucidate its potential mechanisms [13]. We found 18 immune-related genes to predict the prognosis of patients with pancreatic cancer characteristics, proving the validity of the IRGP signature, and validating the model and relationship between the immune cells [14]. Bioinformatics analysis integrates three immune-related long-term noncoding RNA (mRNA, microRNA, lncRNA) signals, which can be used as a predictive model for clear cell renal cell carcinoma [15].

The combination of two biomarkers is better than a single gene [16] regarding the accuracy of cancer diagnosis and prognosis models. For this study, we used a new modeling algorithm, pairing, and iterative operation, to build an irlncRNA signal without any fixed expression level. Finally, we evaluated its prognostic value in patients with colon cancer and its diagnostic efficacy, drug sensitivity, and tumor immune invasion.

## Materials and methods

### Obtaining colon cancer data from TCGA

The transcriptome data of the fragments per kilobase million (FPKM) format and clinicopathological information of colon cancer were downloaded from TCGA (https://portal.gdc.cancer.gov/). The data were derived from 41 normal patients and 373 tumor patients. Ensembl (http://asia.ensembl.org) was used to download the gene transfer format (GTF) file to differentiate mRNAs from lncRNAs for subsequent analysis. Immune genetic databases (http://www.immport.org) were used to download immune-related genes and were coexpressed to screen irlncRNAs. The correlation between immune genes and all lncRNAs was analyzed. The absolute value of the immune gene correlation coefficient was greater than 0.4, and the *p* value was less than 0.001. To determine the DEirlncRNAs, we used the limma R package related to immune lncRNA differential expression analysis. The threshold was set to a log fold change (FC) > 2 and false discovery rate (FDR) < 0.05.

### Pairing DEirlncRNAs

If the expression quantity of lncRNA A is lower than that of lncRNA B, C is defined as 0; otherwise, C is defined as 1. Next, the constructed 0 or 1 matrix continued to be filtered. When the number of lncRNAs with 0 or 1 expression was more than 20% and less than 80% of the total, it was considered an effective match. If the expression level of an lncRNA pair is 0 or 1, the relationship between the lncRNA pair and survival prognosis is not considered because no specific level of lncRNA pairs can correctly predict the survival outcome of patients.

### Build risk model to assess risk score

First, univariate factor analysis was performed, and then lasso regression was executed to screen immune gene pairs. In lasso regression, 1000 cycles were run. For each loop, random stimulation was set to 1000 times, and the frequency of each pair in 1000 repetitive lasso regression models was recorded. Next, cross-validation was performed. Finally, the immune pairs were analyzed by Cox proportional hazards regression, and the model was constructed. The AUC values of each model were calculated and plotted. The larger was the AUC, the higher was the accuracy of predicting survival. Given that the curve reached the zenith, indicating that the AUC value was the largest, the calculation process was terminated, and the model was deemed the optimal candidate. The 1-, 2-, and 3-year ROC curves of the model were drawn. The established risk model was used to calculate the risk score of all clinical cases. Risk score = ${\varSigma}_{i=1}^n Coe{f}_i\times {x}_i$, n, *x*_*i*_ and *Coⅇf*_*i*_ represents the number of genes, gene expression level, and coefficient value respectively. The AIC value of each point in the 3-year ROC curve was calculated to determine the maximum inflection point, which had the best specificity and sensitivity.

### Risk assessment model validation

To demonstrate this cutoff point, we performed Kaplan–Meier analysis and visualized the survival curve to show the distinction in survival between low-risk and high-risk groups. The risk scores of all the samples in the risk model were visualized using the R tool. The R packages used in these steps include survival, glmnet, surviviner, survivalROC, pbapply, and P Heatmap. Additionally, to prove the clinical application value of the model, we used chi squared test to analyze the connection between the risk model and clinicopathological characteristics. The results of the analysis are displayed in the strip diagram, marked as described below: * < 0.05,** < 0.01, *** <0.001. The Wilcoxon signed-rank test was used to calculate the difference in the risk score between groups with different clinicopathological characteristics. Block diagram visualization was used to show the analysis results. To determine whether the risk model can be used as an independent predictor of clinical prognosis, univariate and multivariate Cox regression analyses were compared between the risk score and clinicopathological features. Forest map visualization was used to demonstrate the results. The R tools used in these operations are ggupbr, p-heatmap, and survival.

### Correlation analysis of tumor-infiltrating immune cells

To identify the relationship between immune cell characteristics and risk scores, we used the following six credible methods to analyze the immune cell infiltration status of patients with colon cancer from the TCGA database: TIMER, CIBERSORT, EPIC, MCPCOUNTER, XCELL, and CIBERSORT. Next, the difference in the content of immune infiltrating cells between the high-risk and low-risk groups was analyzed using the Wilcoxon signed-rank test. Spearman correlation analysis was used to analyze the relationship between the risk score and immune infiltrating cells. The correlation coefficient of the results showed that in the lollipop diagram, the implication threshold was set as *p* < 0.05. The program was executed using the rggplot2 package.

### To explore the significance of the model in clinical treatment

To evaluate the application of this model in the clinical treatment of colon cancer, we calculated the IC50 of common administrating chemotherapeutic drugs for colon cancer patients in TCGA. The Wilcoxon signed-rank test was used to compare the IC50 differences between the high-risk and low-risk groups. The results are shown in the block diagram, which was obtained using the prrophetic and ggplot2 packages in R.

### Model- and immune checkpoint inhibitor-related genes

To evaluate the risk model using the immune checkpoint inhibitor-related gene expression level, we used the ggstatsplot package for data visualization.

## Result

### Extraction of DEirlncRNA

**Fig. 1:**
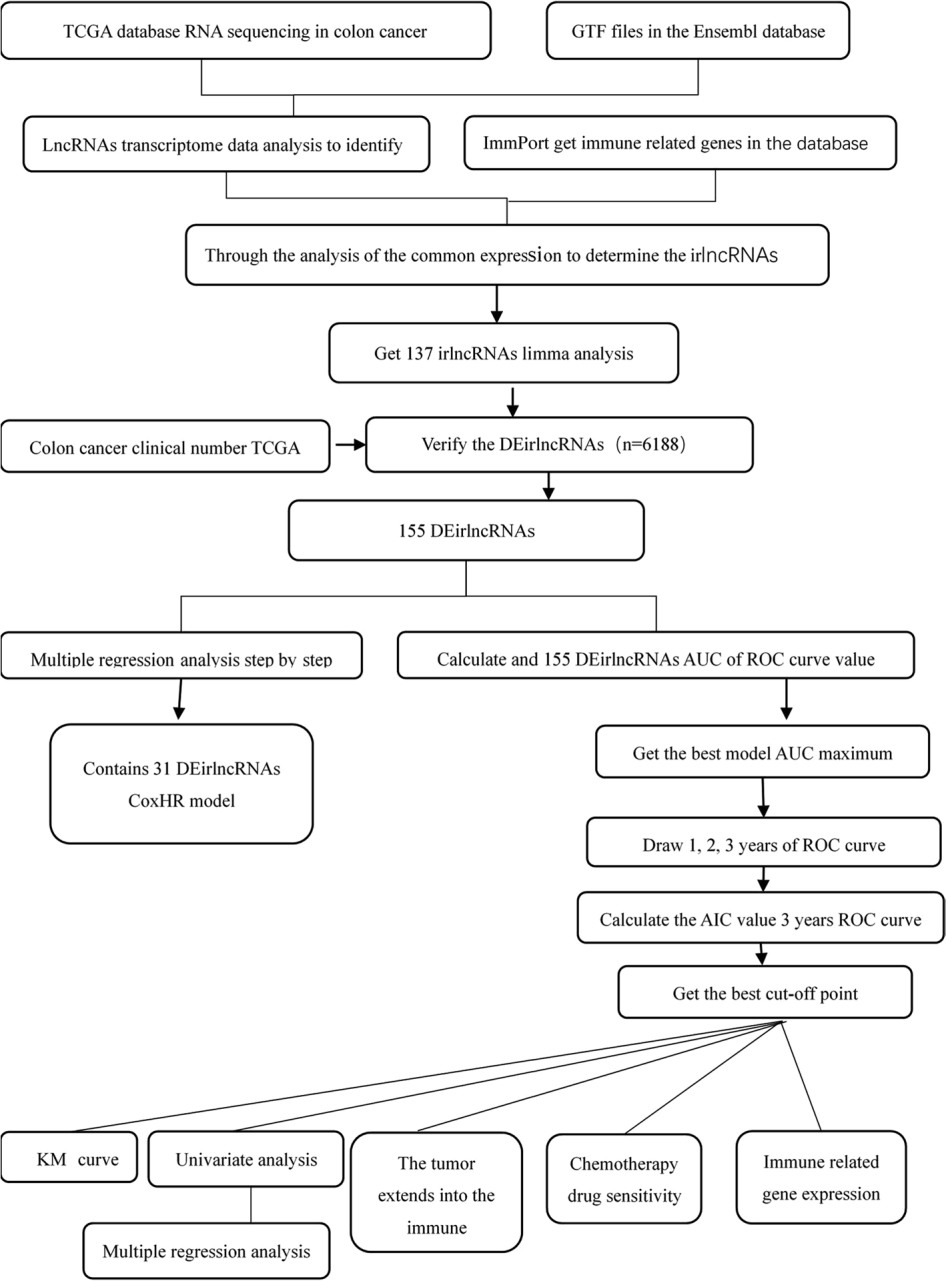
Flow chart of this study

**Fig. 2:**
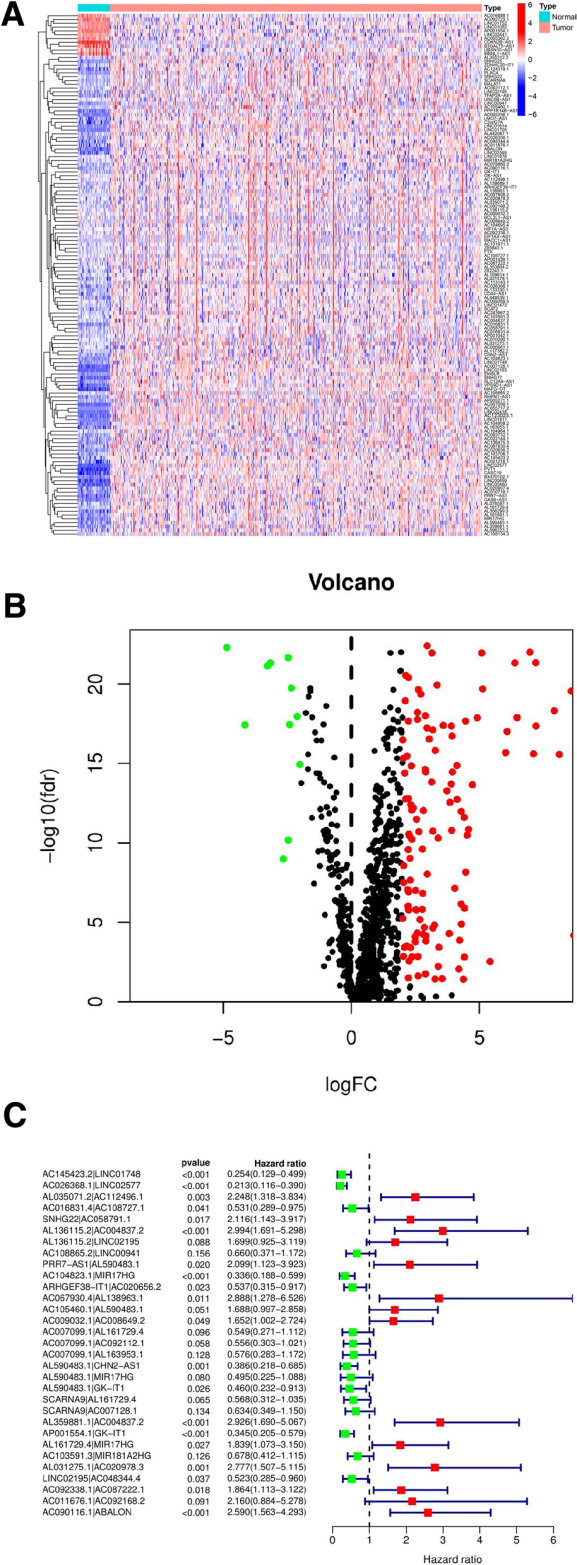
Extract has the difference of immune-related genes. Using the TCGA data set and immune-related Ensembl annotation to identify differentially expressed genes. Shows the heat map figure (**A**) and the volcano (**B**). (**C**) The forest figure shows the COX proportional hazards regression method of stepwise regression method and the DEirlncRNAs

The process is shown in Fig. 1. We retrieved and downloaded the transcriptome and clinical datasets of colon cancer from the colon cancer project of the TCGA database including 41 normal samples and 473 tumor samples. Next, the data were annotated according to the GTF file of Ensembl to distinguish mRNA from lncRNA. The immune genes were downloaded from the immune report database, and coexpression analysis was executed between the known immune genes and lncRNAs. We identified 1093 irlncRNAs (Suppl. Fig. 1); 137 were DEirlncRNAs (Fig. 2A), 126 were upregulated, and 11 were downregulated (Fig. 2B)**.**

### Construction of the immune prognosis model and risk prediction model

**Fig. 3:**
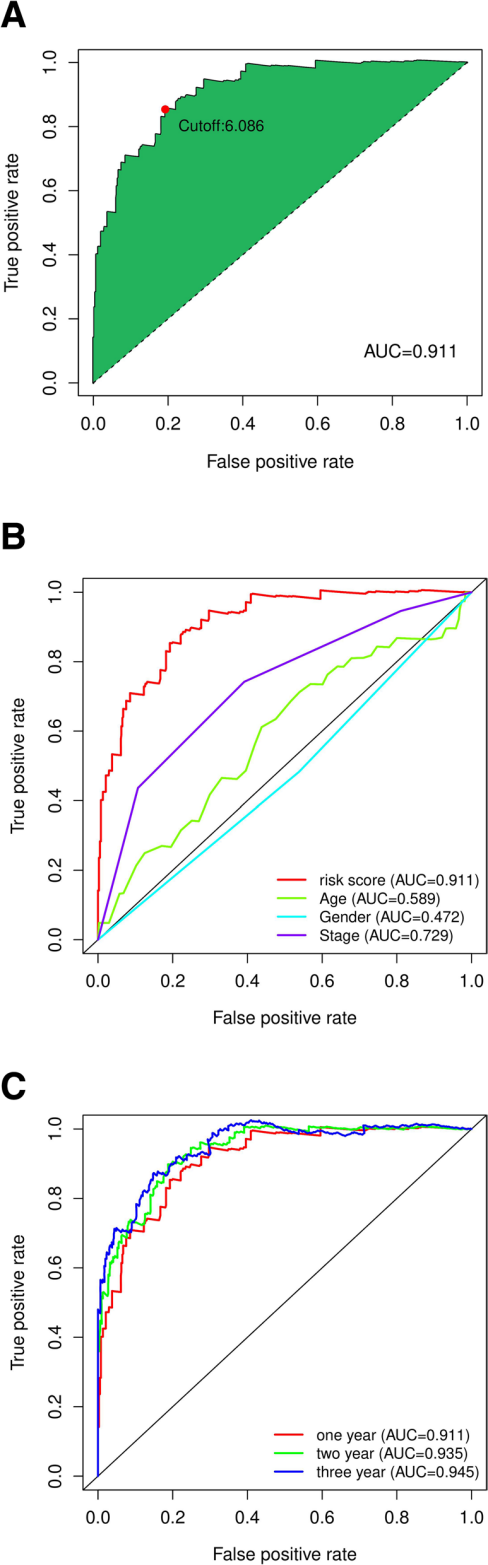
Immune-related genes were used to establish a risk assessment model. (**A**) Draw 155 DEirlncRNA each generated on the model of ROC AUC value curve, and the highest point of the AUC was determined. ROC ideal DEirlncRNA model associated with the AUC value of maximum. Of 426 patients with colon cancer risk score, the biggest inflection point is made by AIC cutoff point. (**B**) 3 years compared with other common clinical features of ROC curve shows the superiority of risk score. (**C**) The optimal model of the ROC 1 year, 2 years, and 3 years shows that all the AUC values are greater than 0.91

Making iterative cycle of 0 or 1 matrix extract 137 DEirlncRNA, 6188 effective DEirlncrna pairs were identified. After univariate testing and improved lasso region analysis, 155 pairs of irlncRNAs were obtained, among which 31 pairs were involved in the Cox risk assessment model in a step-by-step manner (Fig. 2 C). We counted the AUC of each subject operating characteristic curve of 155 pairs of subjects, drew the curve, and calculated a value of 0.911. We also used the AIC value to identify the maximum inflection point as the cutoff point of the 3-year ROC curve, collected the data of 426 patients with acceptable colon cancer from TCGA for the risk score, and established the cutoff point to redifferentiate the low-risk and high-risk groups in the cohort for testing (Fig. 3A). The 3-year ROC curve was compared with other clinical data (Fig. 3B). To verify the optimality, we plotted ROC curves for 1, 2, and 3 years, indicating that all the AUC values exceeded 0.91 (Fig. 3C).

### Clinical correlation analysis based on the risk prediction model

**Fig. 4:**
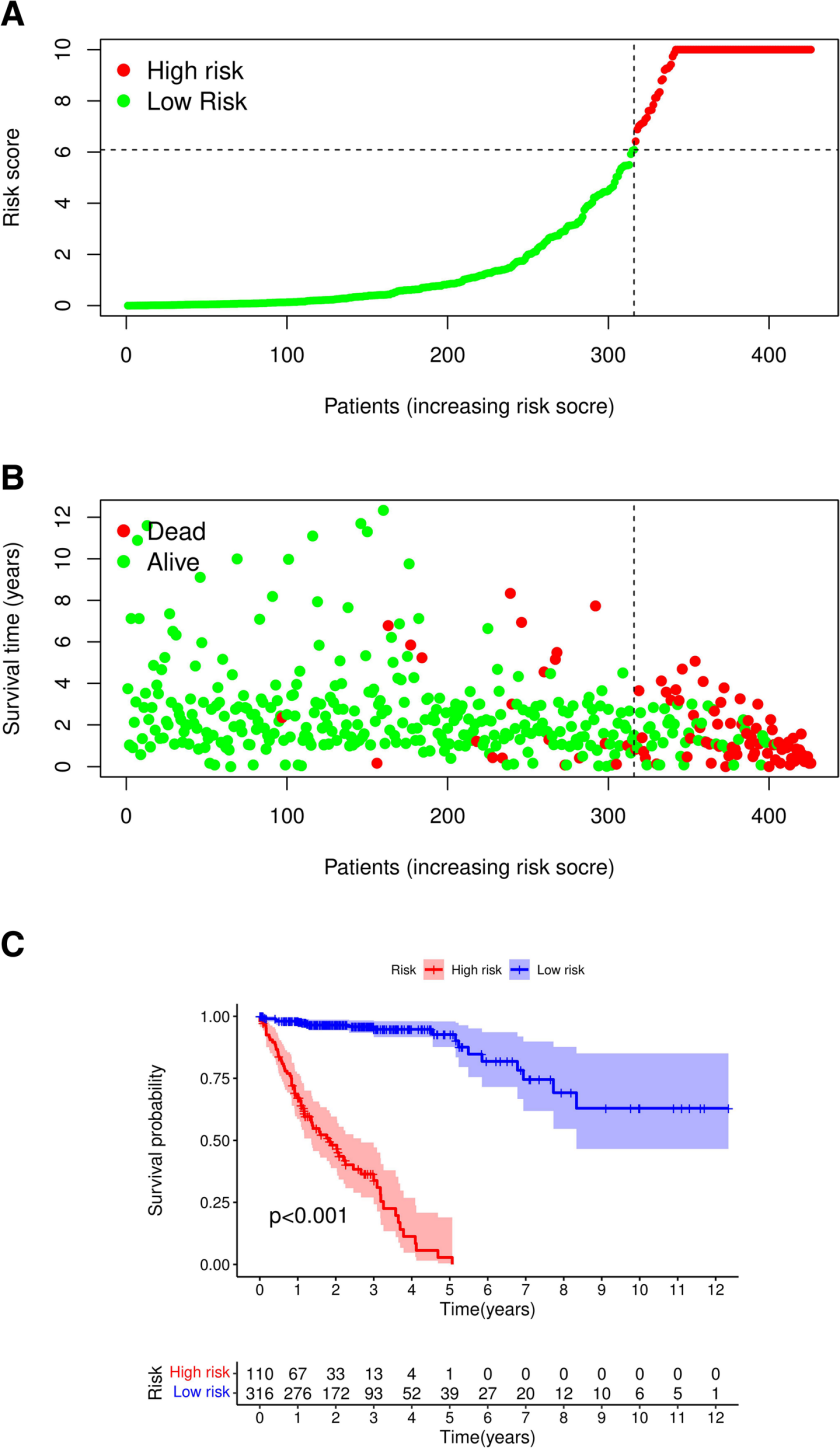
Risk assessment model of survival prognosis. (**A**) and (**B**) shows risk score and survival results of each case. (**C**) By the Kaplan-Meier test, high-risk group of patients’ survival time is short

**Fig. 5:**
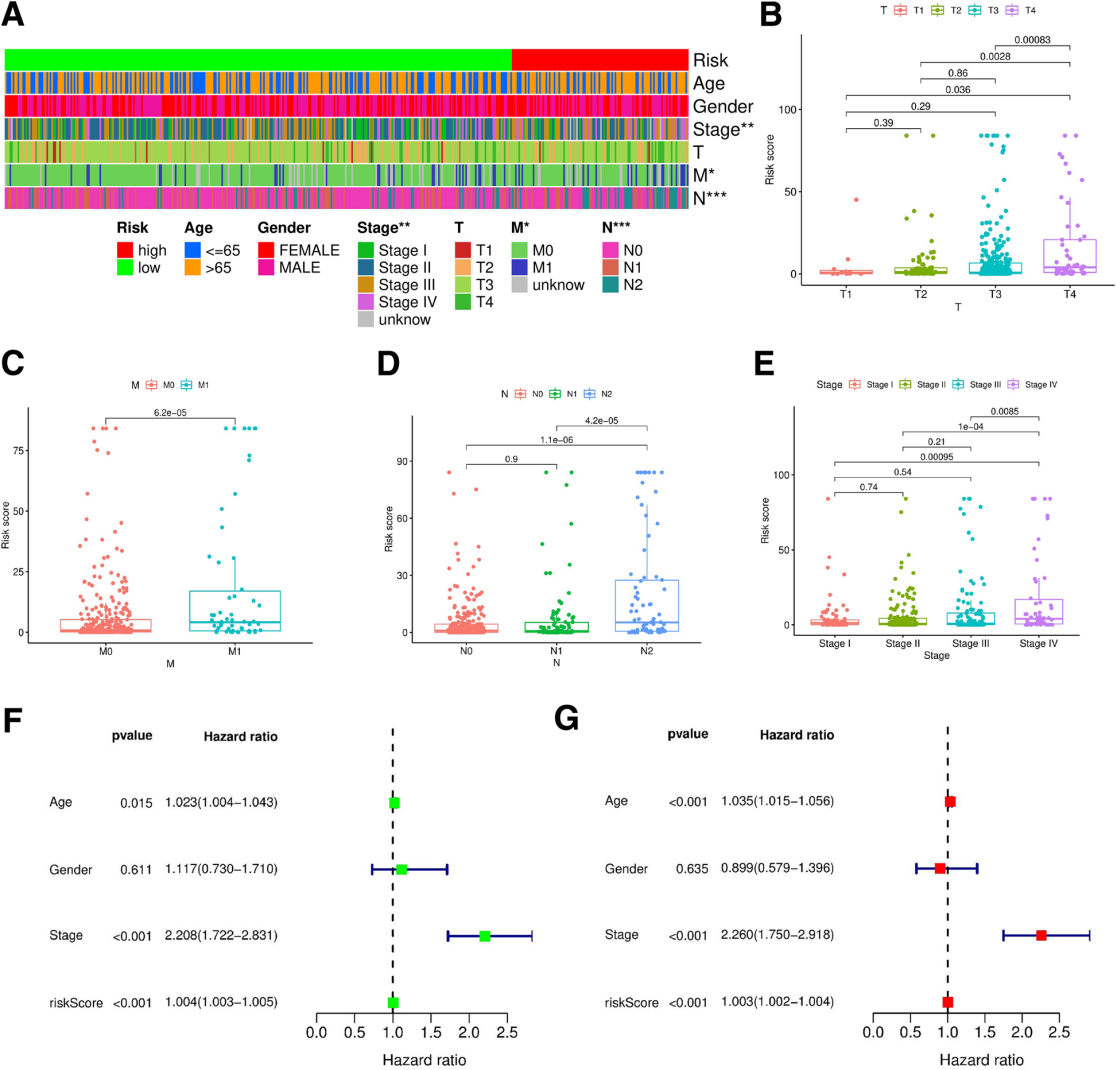
clinical correlation analysis based on risk assessment model. (**A**–**G**) Bar graph (**A**) and scatter diagram show that *T* (**B**), *M* (**C**), *N* (**D**), and (**E**) clinical stage were associated with a significant risk score. (**F**) Single-variable Cox regression to prove the clinical stage (*p* < 0.001, HR = 2.208, 95% CI [1.722–2.831]), the risk score (*p* < 0.001, HR = 1.004, 95% CI [1.003–1.005]) showed statistical significance, and multivariate Cox regression analysis prove that age (**G**) (*p* < 0.001, HR = 1.035, 95% letter interval [1.015–1.056]) and the clinical stage (*p* < 0.001, HR = 2.260, 95% CI [1.750–2.918], HR = 1.003, 95% CI [1.002–1.005])

Based on the cutoff point of AUC determined before the risk assessment model for clinical relevance evaluation, 110 subjects were classified as the high-risk group, and 316 patients were classified as the low-risk group. The risk score and survival are shown in Fig. 4A and Fig. 4B, respectively. These data suggest that with increasing value at risk, the death toll rises. Kaplan–Meier analysis showed that the survival time of patients in the high-risk group was shorter than that of patients in the low-risk group (*p* < 0.001) (Fig. 4C). Next, we used chi-squared test to evaluate the risk of colon cancer and the relationship between the clinicopathological features. The Wilcoxon signed-rank test strip chart (Fig. 5A) and scatter diagram show that T (Fig. 5B), M (Fig. 5C) and N (Fig. 5D) stages and clinical staging (Fig. 5E) were associated with a significant risk. Next, we performed univariate Cox regression analysis (Fig. 5F) and proved that the clinical stage (*p* < 0.001, HR = 2.208, 95% CI [1.722 2.831]) and risk score (*p* < 0.001, HR = 1.004, 95% CI [1.003 1.005]) showed statistical significance, and multivariate Cox regression analysis (Fig. 5G) proved that age (*p* < 0.001, HR = 1.035, 95% CI [1.015 1.056]) and clinical stage (*p* < 0.001, HR = 2.260, 95% CI [1.750 2.918]), HR = 1.003, 95% CI [1.002 1.005]) (the detailed numerical values are in Table 1).

**Table 1:**
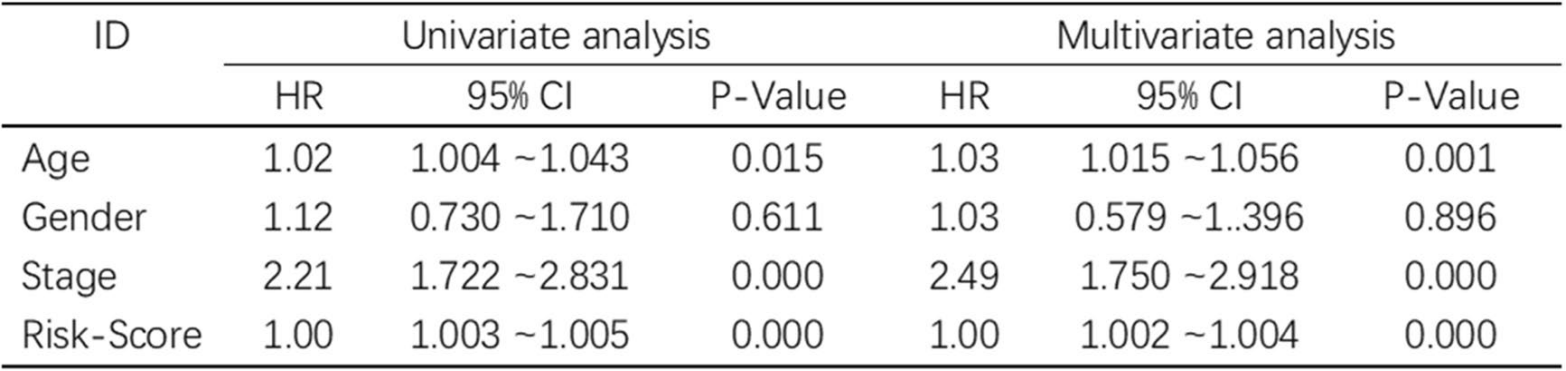
univariate and multivariate Cox regression analysis of the specific value

### A risk assessment model can predict immunosuppressive molecules and tumor-infiltrating immune cells

**Fig. 6:**
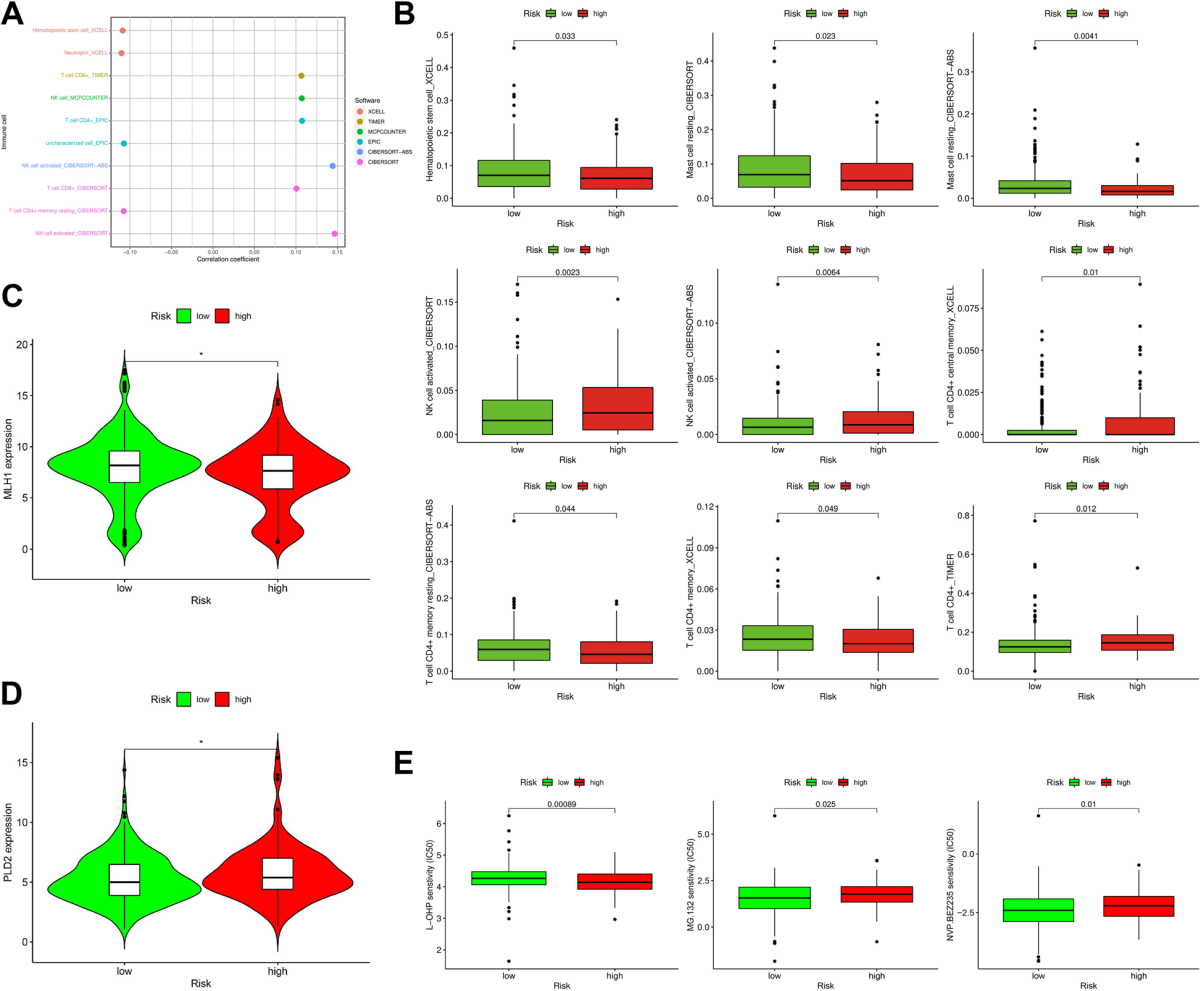
risk assessment model of tumor-infiltrating cells, immune checkpoint inhibitory molecules, and drug sensitivity analysis. (**A**) Spearman’s correlation analysis showed that the high-risk group of patients with CD4 T cells, monocytes, and tumor-infiltrating immune cells were positively correlated, and negative correlation with hematopoietic stem cells and neutrophils. (**B**) Using the risk model to predict tumor-infiltrating immune cells representative results. (**C** and **D**) The risk model is associated with immune checkpoint inhibitor-related biomarkers and found that high-risk score and PLD2 (*p* < 0.05) and high expression were positively related to (**C**), and MLH1 (*p* < 0.05) in the lower expression of negative correlation (**D**). (**E**) The high-risk score with chemotherapy drugs such as oxaliplatin into (*p* = 0.00089) the lower IC50, and the high-risk score is related to the high half-inhibitory concentration (IC50) of the protease inhibitors MG.132 (*p* = 0.025) and NVP-TAE684 (*p* = 0.01)

Because immune-related genes and lncRNAs initially correspond, we evaluated whether the model is relevant to the tumor-immune microenvironment. The high-risk group was negatively correlated with tumor-infiltrating immune cells such as hematopoietic stem cells and neutrophils and positively correlated with CD4 T cells and monocytes. Spearman correlation analysis was performed, and the results are shown in Fig. 6A, B, and Table 2. Immune checkpoint inhibitors were used to treat colon cancer in clinical medication. We studied whether the risk model is relevant to immune checkpoint inhibitor-relevant biomarkers and found that a high stake score was positively correlated with high expression of PLD2 (*p* < 0.05; Fig. 6C) and negatively correlated with low expression of MLH1 (*p* < 0.05; Fig. 6D).

**Table 2:**
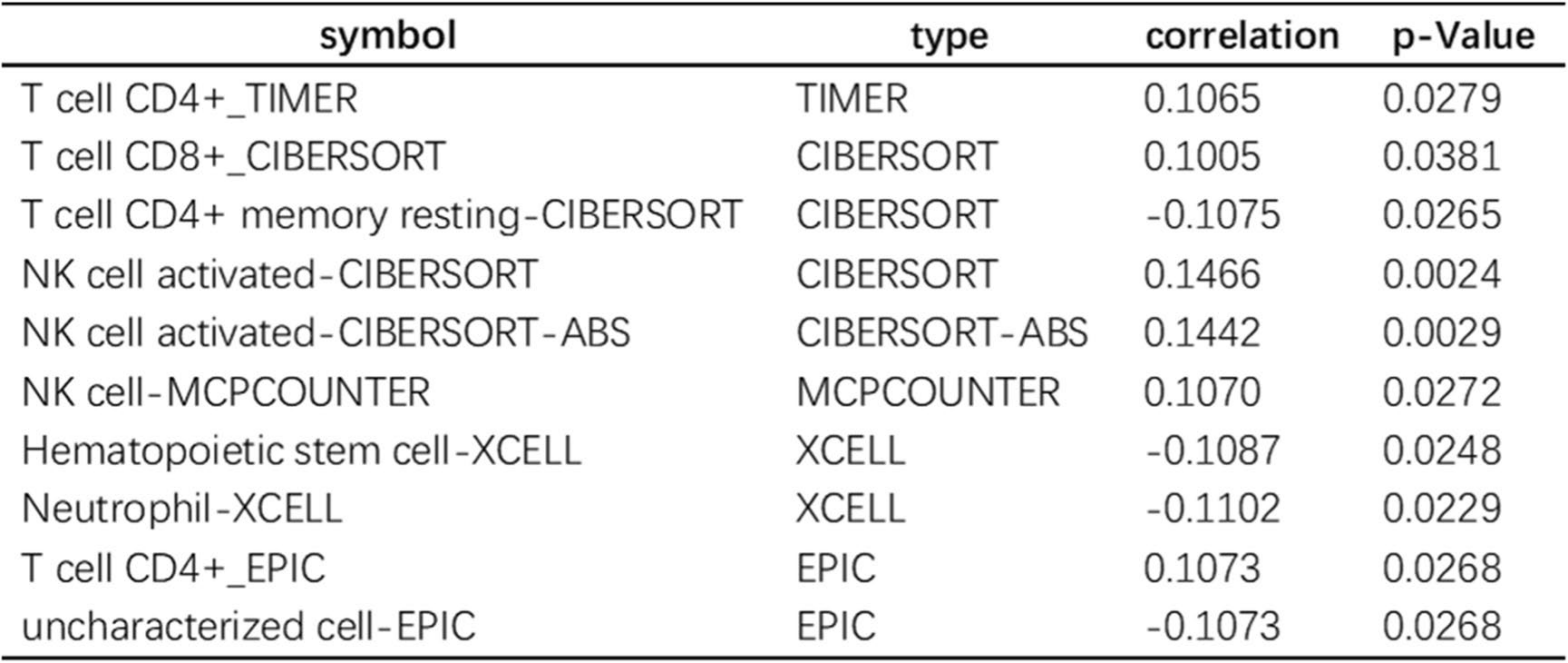
Tumor infiltration of immune cells and risk scores of the comparison results

### Correlation analysis between the risk model and chemotherapy drugs

In addition to checkpoint blockade treatment, we determined the association between the risk and efficacy of common chemotherapy drugs for colon cancer in TCGA colon cancer patient data. We show that high-risk scores are associated with a low IC50 of chemotherapeutic drugs such as oxaliplatin (*p* = 0.00089); however, it is related to the high half inhibitory concentration (IC50) of the protease inhibitor (MG132) and anaplastic lymphoma kinase inhibitor (NVP-TAE684). This model can be used as a potential predictor of chemosensitivity (Fig. 6E).

## Discussion

Surgery is a common treatment for colon cancer, but many shortcomings exist. Recent studies have found that the immune environment of primary tumors has a profound impact on immunotherapy [17]. Pembrolizumab monotherapy has shown long-lasting antitumor activity in non-small-cell lung cancer (NSCLC) expressing advanced programmed death ligand 1 (PD-L1, [18]). Some tumor cells can use SNHG12 to escape immune-mediated attack and enhance the immune response [19]. The expression of mir155HG was significantly correlated with the infiltration level of immune cells and immune molecules [20]. These studies indicate that immune intervention therapy has broad application prospects in tumor therapy. Therefore, new methods of treatment and prognosis of colon cancer must be identified. Additionally, lncRNAs were identified as essential regulators of the cancer immune response. In the current study, enlightened by the tactics of immune-relevant gene pairing, we attempted to build a viable model of double genome pairing.

First, we collected transcriptome data and clinical data and downloaded immune-related gene datasets from TCGA. Differential coexpression analysis was executed to classify the data, and we verified the nonrepetitive sequence pairs using the improved circular single-pair method and 1 or 0 matrix. Next, we performed univariate analysis, combined with the improved lasso penalized regression, to increase the accuracy and effectiveness of risk prediction [21]. We obtained prognosis-related immune-related lncRNAs to construct a prognostic model of immune-related lncRNAs. We not only calculated every AUC value on the ROC curve to obtain the best model but also obtained the AIC value of all points on the AUC curve to identify the best cutoff point, which was used to distinguish the high-risk and low-risk groups in colon cancer patients. Finally, we evaluated the new model in a clinical setting and included the survival rate, clinicopathological features, tumor-infiltrating immune cells, immunosuppressive biomarkers, and chemosensitivity.

The present study explored a signal based on seven irlncRNAs to predict the overall survival rate of colon cancer [22]. Generally, the high expression of lncRNAs shows important biological functions. Our data analysis can identify the DEirlncRNAs and identify the most valuable irlncRNAs. Therefore, detecting the value of each specific irlncRNA was not required, only the high or low expression of irlncRNA pairs. The prediction model can differentiate the high risk and low risk of patients with clinical disease. In this study, some of the DEirlncRNAs in the process of modeling that have been already identified play an important role in malignant phenotypes of various cancer types, such as SNHG22 [23, 24], PRR7-AS1 [25], and LINC00941 [26, 27], especially for colon cancer, while others were revealed for the first time. For instance, SNHG22 promoted CRC tumorigenesis and metastasis by sponging miR-128-3p [28]. Reveal the essential role of LINC00941 [29] in metastatic CRC via activation of the TGF-β/SMAD2/3 axis. Demonstrate that MIR17HG [30] plays an oncogenic role in colorectal cancer. Long noncoding RNA (lncRNA) imbalance has been found in many human cancers, including colon cancer. Therefore, identifying potential lncRNA biomarkers with prognostic value is crucial [31]. Our prediction model can distinguish new markers for later study.

To continue to improve the modeling process, we counted each AUC value to determine the peak value of the best model and then compared it with related clinical data. We used the AIC value to determine the best cutoff point for model fitting. The ROC curve shows that the risk score can provide a better AUC than other clinical features. Second, the connection between the risk assessment score and clinical pathology was analyzed. After using this prognostic model to distinguish the low-risk group from the high-risk group, we reappraised the survival results, performed multivariate and univariate analyses of the clinicopathological characteristics, and explored tumor cell immune infiltration, chemotherapeutic drug sensitivity to colon cancer, and biomarkers related to immunosuppressive agents, indicating that this model is useful for the treatment and prognosis analysis of colon cancer.

According to the current research findings, immune cell infiltration can be used as a biomarker for the diagnosis and prognosis of stage I–III colon cancer [32]. To assess the connection between the risk score and tumor-infiltrating immune cells, we used six common methods to evaluate immune infiltrating cells—TIMER [33], CIBERSORT [34], XCELL [35, 36], MCPCOUNTER [37], EPIC [38], and CIBERSORT-ABS [39]. The results showed that lncRNAs with different expression levels were positively correlated with tumor-infiltrating immune cells such as CD4+ T cells, CD8+ T cells, and NK cells but negatively correlated with hematopoietic stem cells and neutrophils. Studies have explained that immune risk scores based on immunohistochemical analysis can demonstrate the therapeutic benefits of immunotherapy and chemotherapy [40]. Stromal cell PD-L1 inhibits the CD8+ T cell antitumor immune response and promotes colon cancer. The MiR-448 targets ido1 and regulates the CD8+ T cell response in human colon cancer [41, 42]. NKILA lncRNA promotes tumor immune escape by sensitizing T cells to activation-induced cell death [43]. Our drug sensitivity analysis indicates that high risk is related to sensitivity to oxaliplatin, MG132, and NVP-TAE684 chemotherapy. Oxaliplatin added to the fluorouracil and folic acid regimens improves adjuvant therapy for colon cancer [44]. MG132 is a potential therapeutic and preventive agent for cancer cachexia [45]. NVP-TAE684 is an anaplastic lymphoma kinase (ALK) inhibitor that inhibits the proliferation of human pancreatic adenocarcinoma cells [46]. We hypothesized that immunotherapy is more beneficial than chemotherapy by removing cancer cells, producing more new antigens, and inhibiting tumor progression. According to the model, we calculated immunosuppressant-related biomarkers such as PLD2 and MLH1. MMR gene (dMMR) deficiency, usually hMSH2 or hMLH1, promotes the development of colon cancer because of mutation or silencing [47]. MLH1 deletion induces the activation of Her-2/PI3K/Akt signaling and leads to cetuximab resistance in colon cancer [48]. The phospholipase D (PLD) family is widely expressed in cells. PLD2 is mainly found on the plasma membrane under nonstimulated conditions. PLD2 can also be used as a downstream effector of various cell surface receptors to trigger and regulate the transmission of intracellular signals during tumorigenesis and metastasis [49]. PLD2 is abnormally expressed in various cancers. Inhibition or elimination of PLD activity has been proven to reduce tumor growth and metastasis [50]. Immunotherapy targeting the ICIs PD-1 and PD-L1 has been widely defined as ICIS, showing an obvious improvement in lifetime, and the combination of immunomodulatory therapy can be further optimized to better block the immunosuppressive pathway of the TME and stimulate antitumor immunity [51].

However, this study has shortcomings. For example, our data were only derived from the TCGA database. However, the model must collect clinical cases and increase the sample size for experimental demonstration because the expression level of different samples may make the model unreliable. We hope that further experiments can be performed in the future, such as Western blotting or drug sensitivity tests.

## Conclusion

irlncRNAs have independent prognostic meaning in colon cancer. Our findings offer a way to predict the prognosis and survival of patients with colon cancer and help to identify which colon cancer patients are more suitable for antitumor immunotherapy. At the same time, our study provides a reference to evaluate other tumor prognosis models.

## Supplementary Information

**Additional file 1: Table S1.** A total of 1093 immune-related lncRNAs were identified by co-expression analysis.

**Additional file 2: Figure S2.** (A) and (C) train queue lasso region, (B) and (D) test queue lasso region, (E), train queue forest map shows 14 DEirlncrna pairs determined by Cox proportional risk regression in the stepwise method, and (F) test queue forest map shows 13 DEirlncrna pairs determined by Cox proportional risk regression in the stepwise method. **Figure S3.** (A) Train queue Risk Score for 213 patients with COAD. the maximum inflection point is the cut-off point obtained by the AIC. (B), Test queue Risk Score for 213 patients with colon cancer. the maximum inflection point is the cut-off point obtained by the AIC. (C) The comparison of the 3-year ROC curve of the train cohort with other common clinical features shows the superiority of risk score. (D) The comparison of the 3-year ROC curve of the test cohort with other common clinical features shows the superiority of risk score. (E) Analysis of time-dependent receiver operating characteristic (ROC) curve in train queue. (F) Time dependent ROC curve analysis of test queue. Figure S4: The prognostic model was validated in the train and test cohorts. (A) Overall survival (OS) of the train queue. (B) Total lifetime of test queue. (C) Risk score distribution in the train queue. (D) Risk score distribution in test queue. (E) Scatter diagram of survival status of train queue. (F) Scatter diagram of survival state of test queue.
