# Supplementary material for: Construction of a new immune-related lncRNA model and prediction of treatment and survival prognosis of human colon cancer
Source: World J Surg Oncol. 2022 Mar 6;20:71. doi: 10.1186/s12957-022-02508-2 (PMC8900415; doi:10.1186/s12957-022-02508-2)
Supplement: Supplementary file 2 — Additional file 2: Figure S2. (A) and (C) train queue lasso region, (B) and (D) test queue lasso region, (E), train queue forest map shows 14 DEirlncrna pairs determined by Cox proportional risk regression in the stepwise method, and (F) test queue forest map shows 13 DEirlncrna pairs determined by Cox proportional risk regression in the stepwise method. Figure S3. (A) Train queue Risk Score for 213 patients with COAD. the maximum inflection point is the cut-off point obtained by the AIC. (B), Test queue Risk Score for 213 patients with colon cancer. the maximum inflection point is the cut-off point obtained by the AIC. (C) The comparison of the 3-year ROC curve of the train cohort with other common clinical features shows the superiority of risk score. (D) The comparison of the 3-year ROC curve of the test cohort with other common clinical features shows the superiority of risk score. (E) Analysis of time-dependent receiver operating characteristic (ROC) curve in train queue. (F) Time dependent ROC curve analysis of test queue. Figure S4: The prognostic model was validated in the train and test cohorts. (A) Overall survival (OS) of the train queue. (B) Total lifetime of test queue. (C) Risk score distribution in the train queue. (D) Risk score distribution in test queue. (E) Scatter diagram of survival status of train queue. (F) Scatter diagram of survival state of test queue. [file 12957_2022_2508_MOESM2_ESM.docx]

**Train Test**

Figure S2A Figure S2B


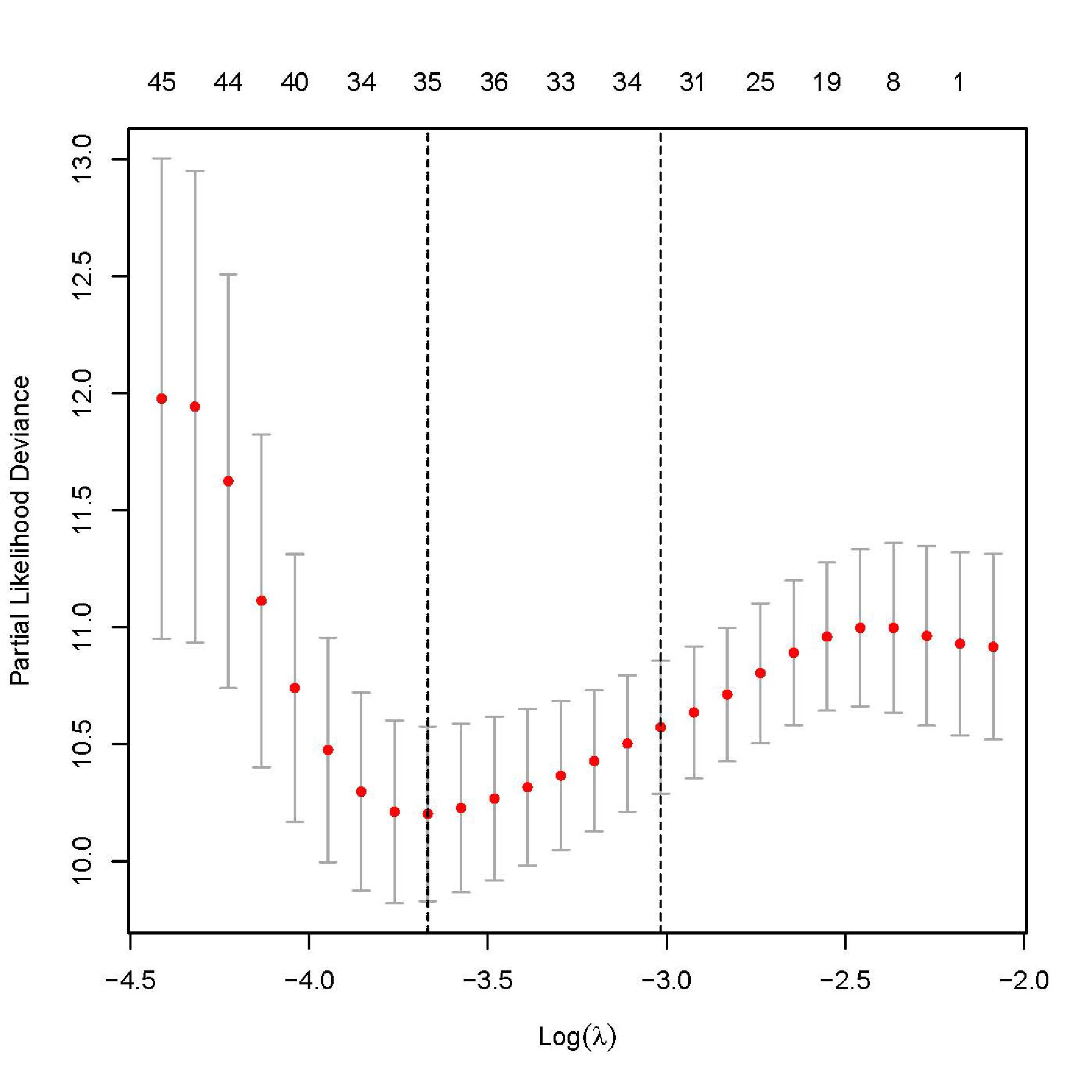

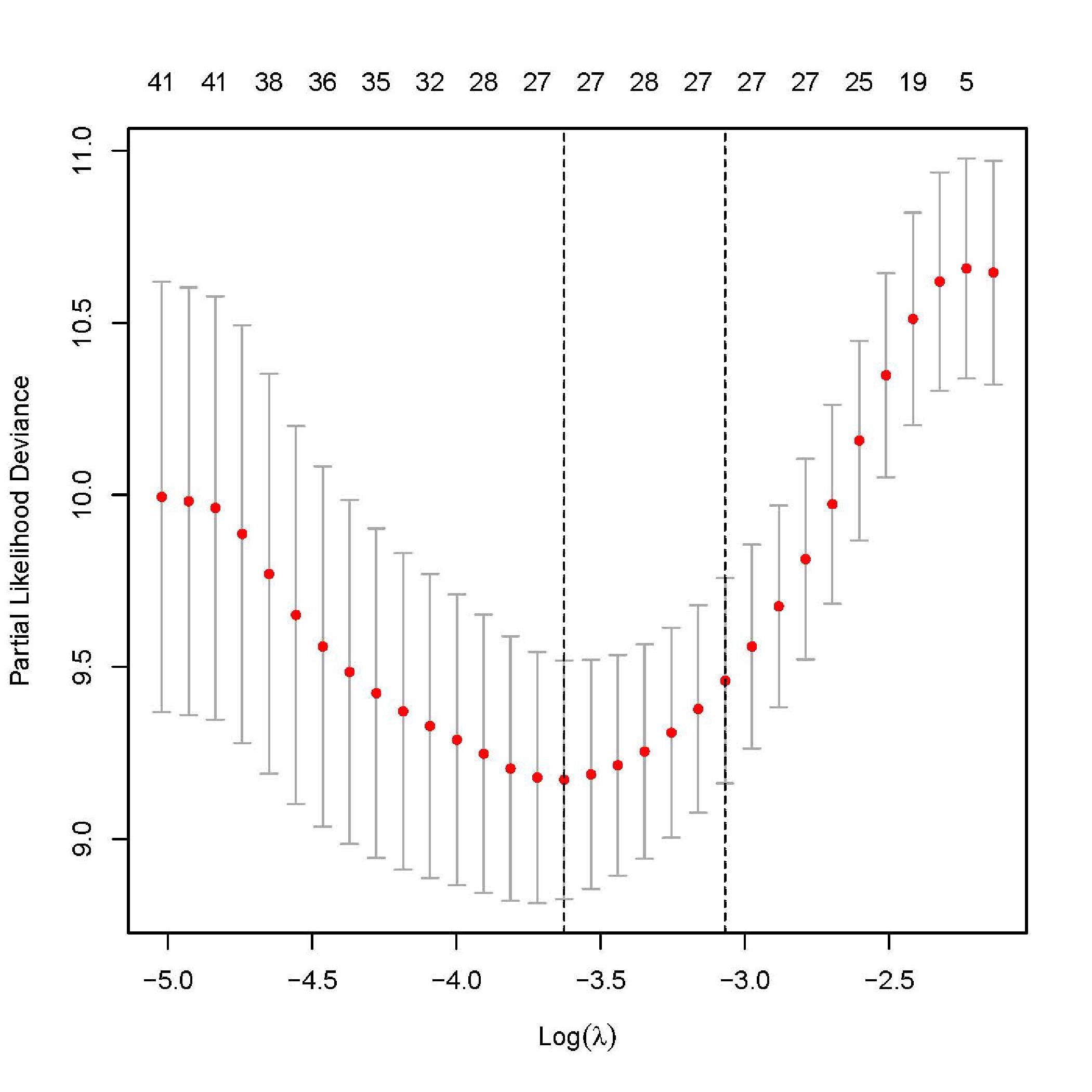


Figure S2C Figure S2D


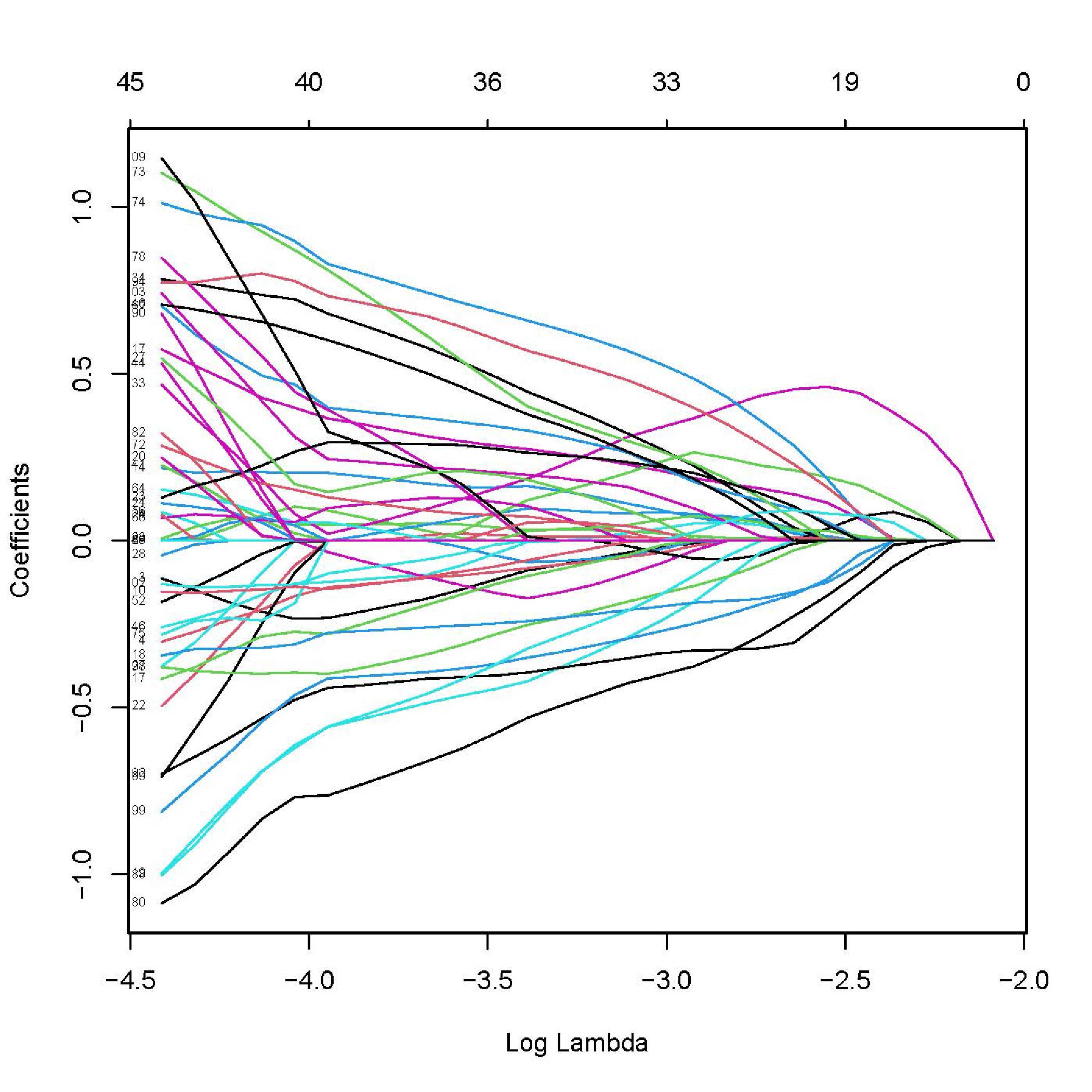

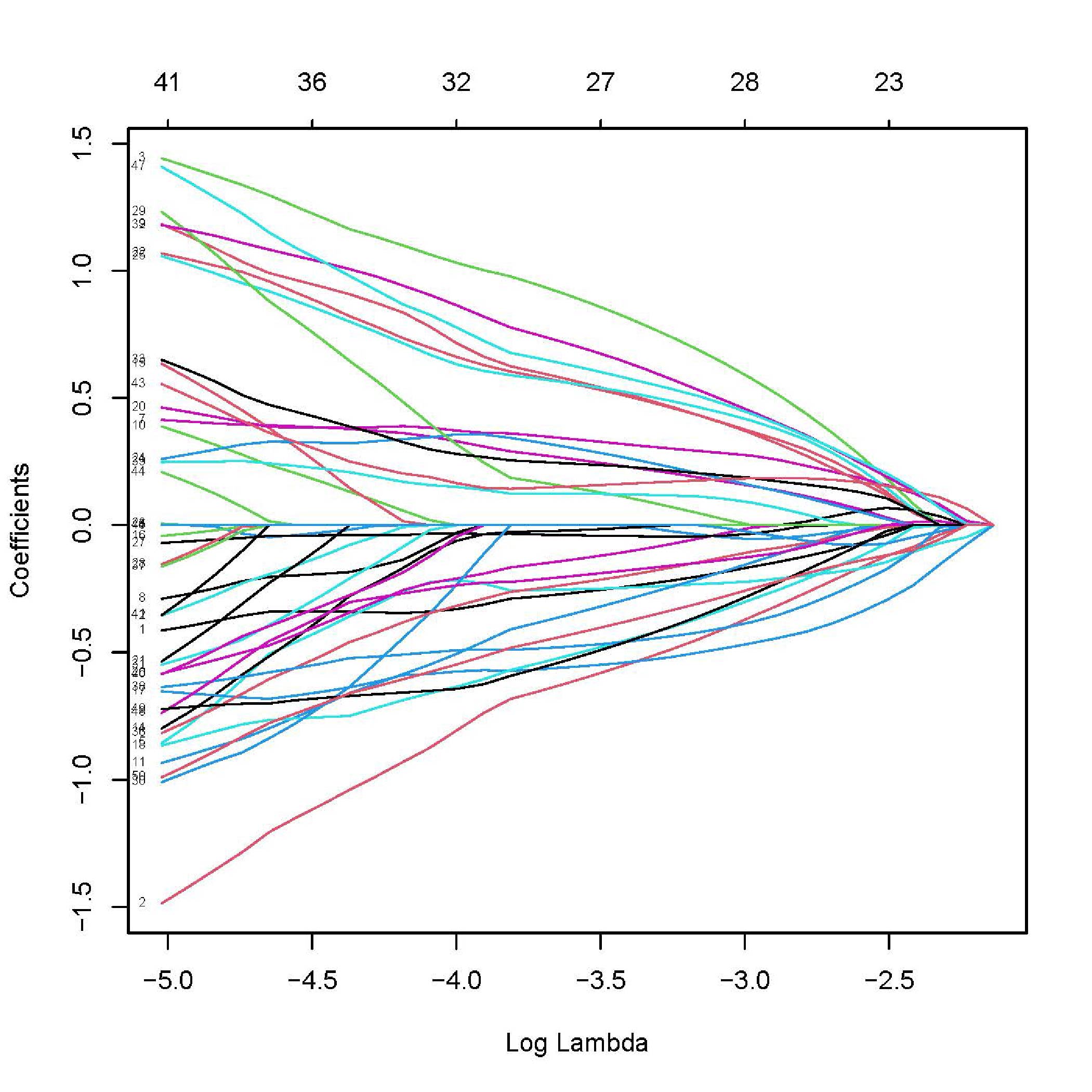


Figure S2E Figure S2F


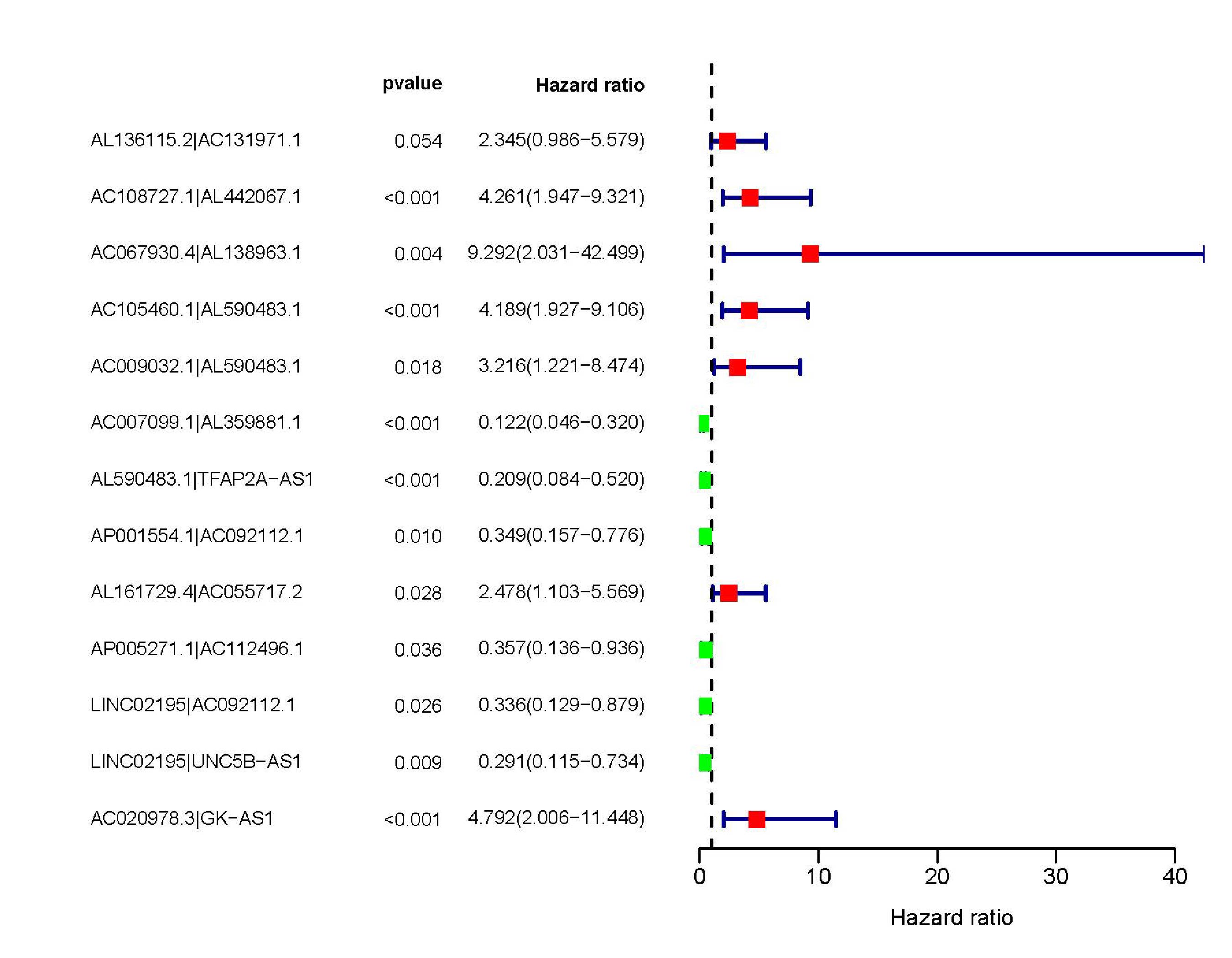

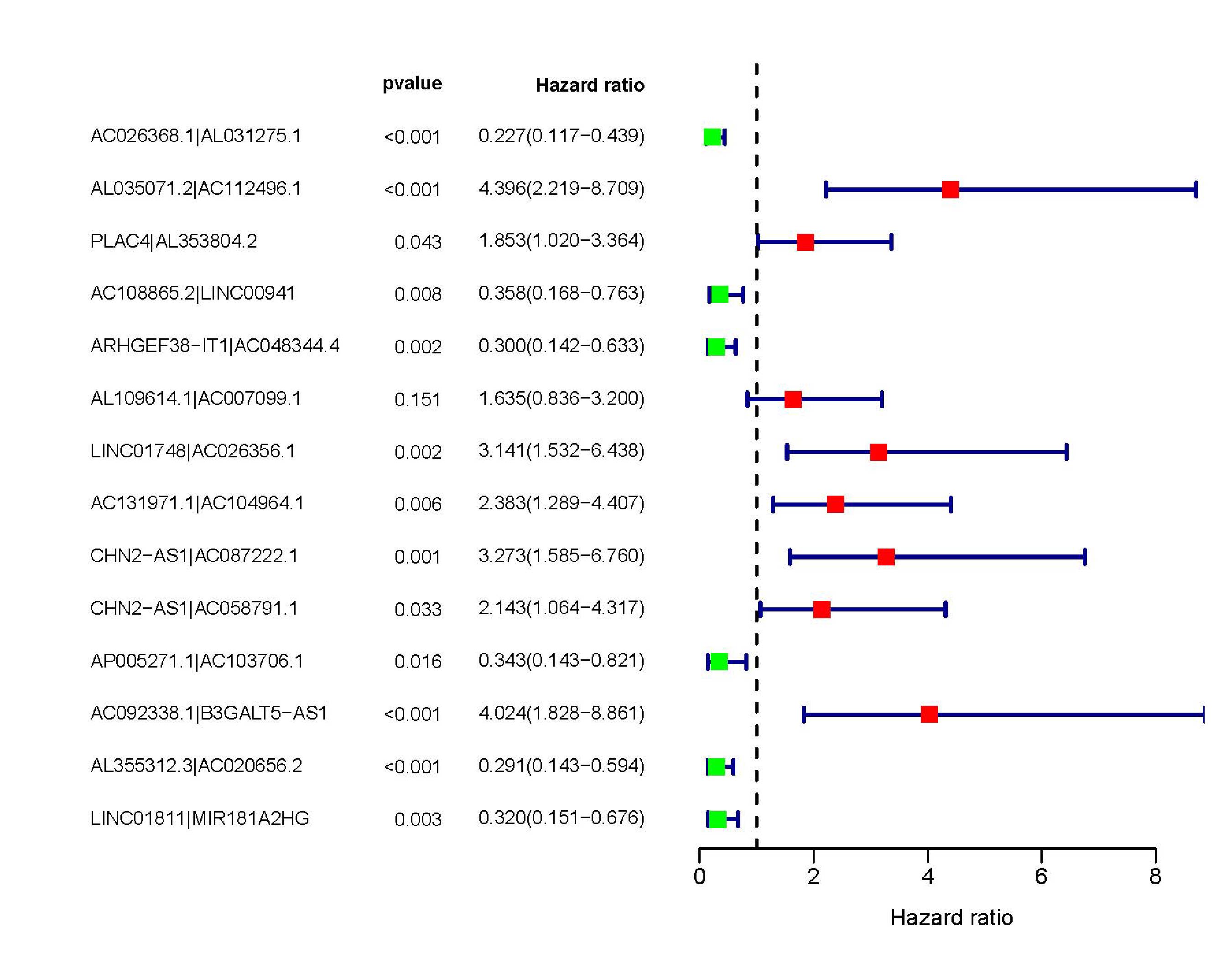


Figure S3A Figure S3B


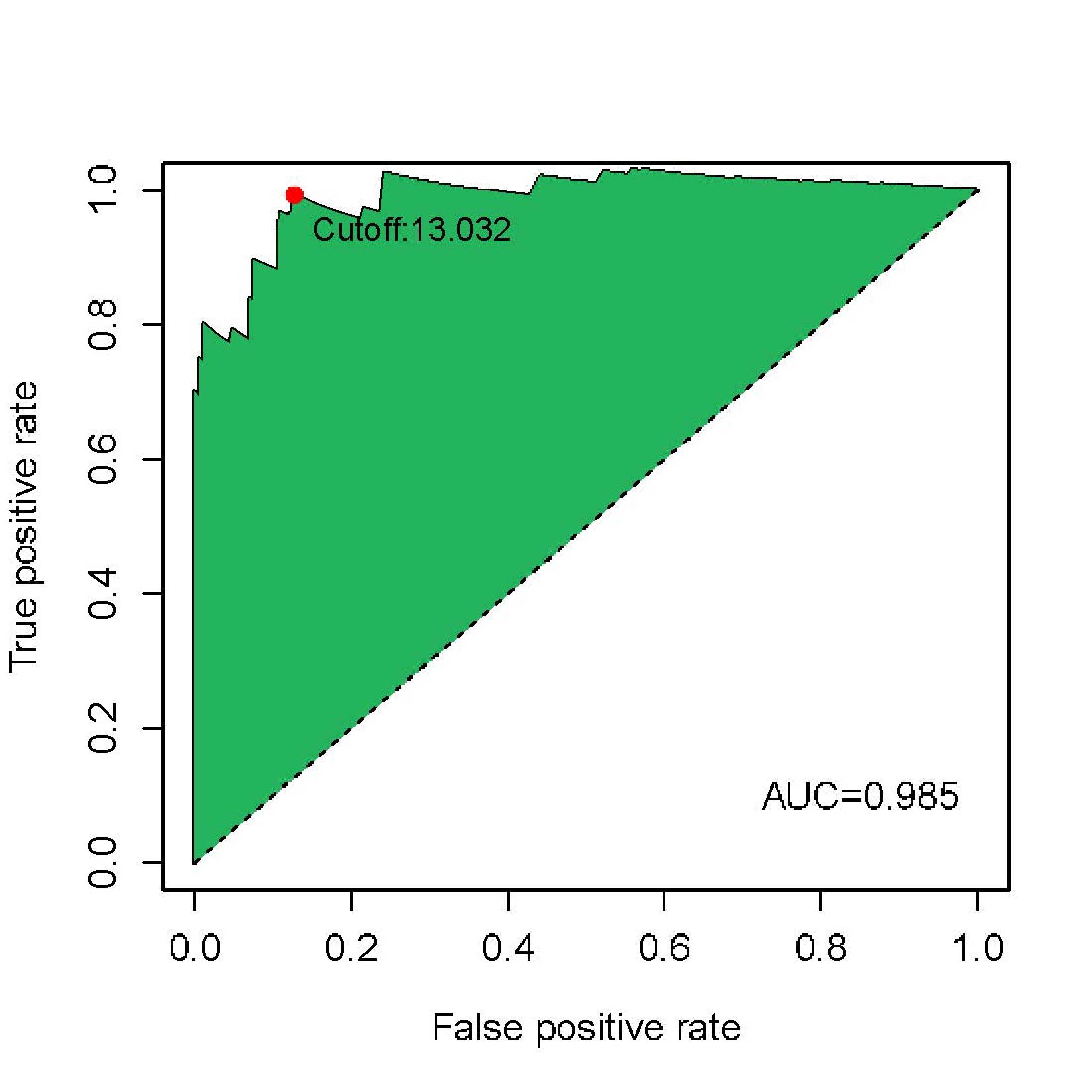

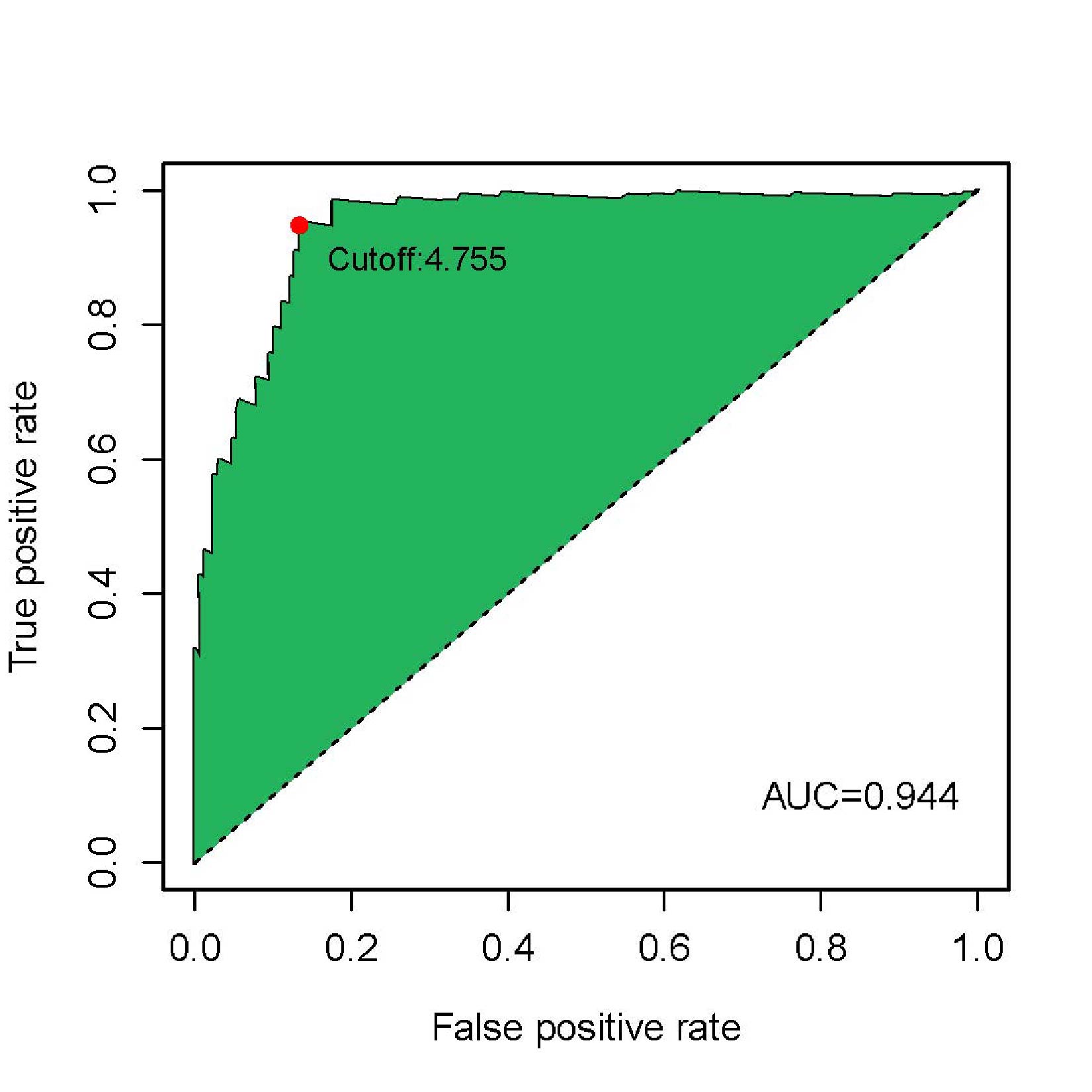


Figure S3C Figure S3D


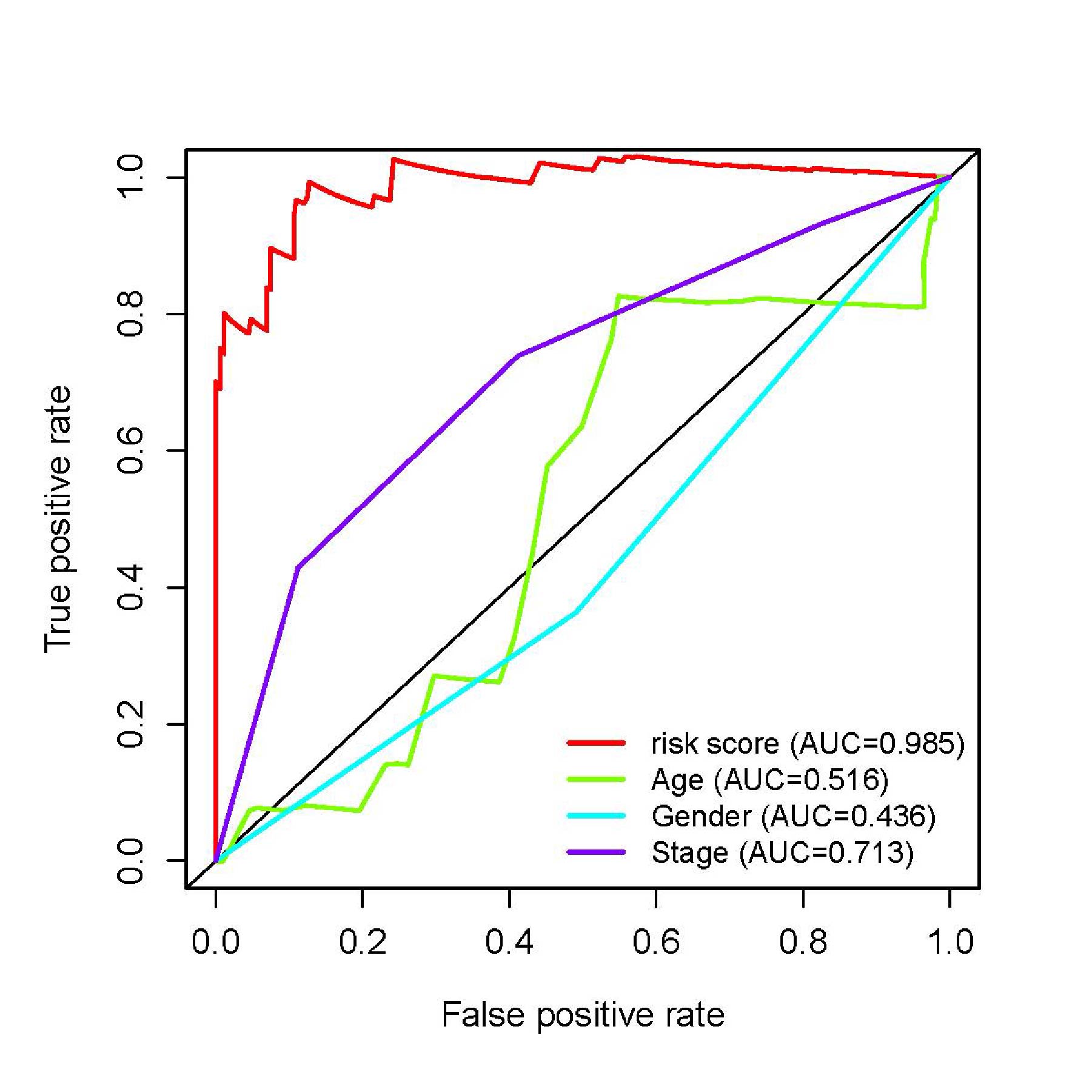

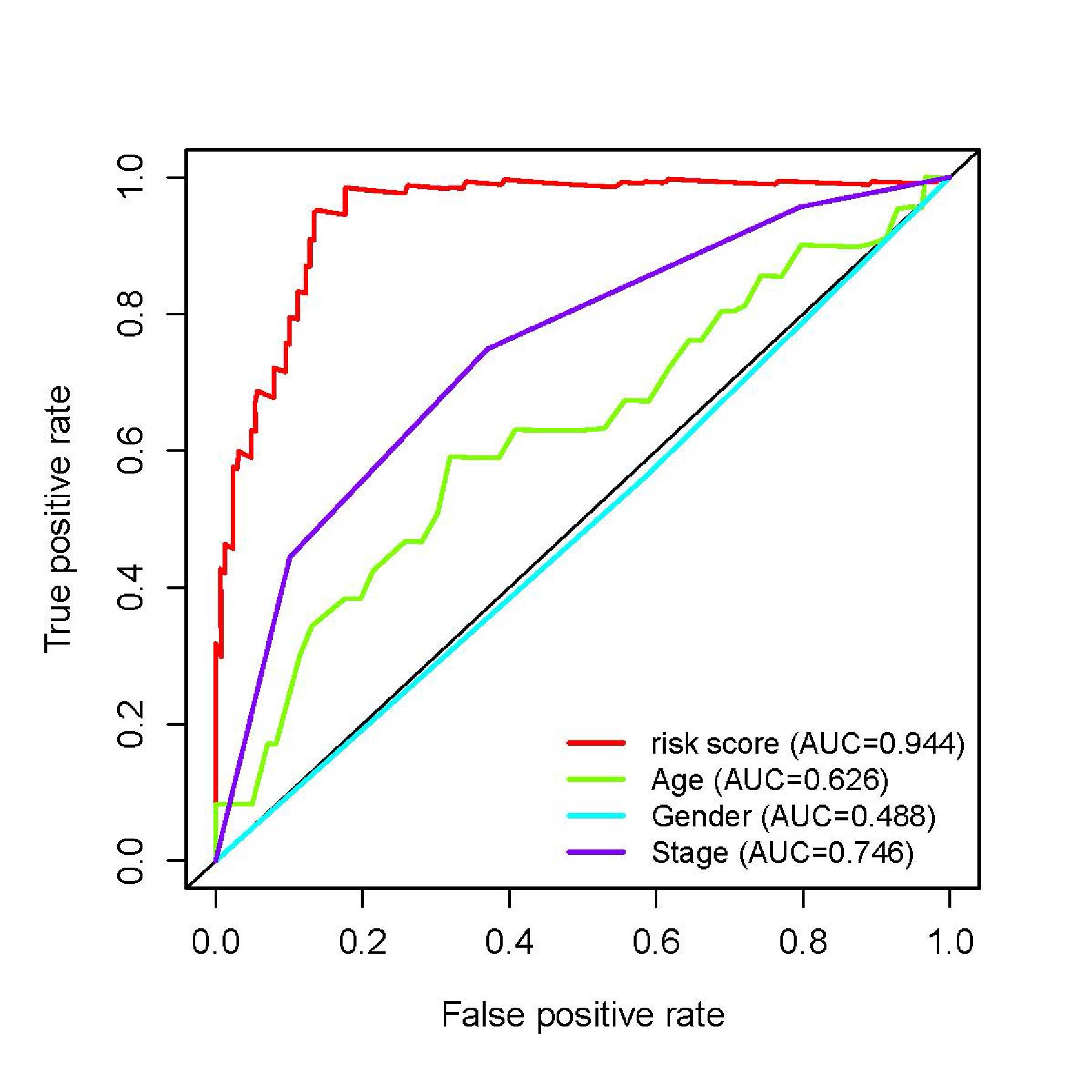


Figure S3E Figure S3F


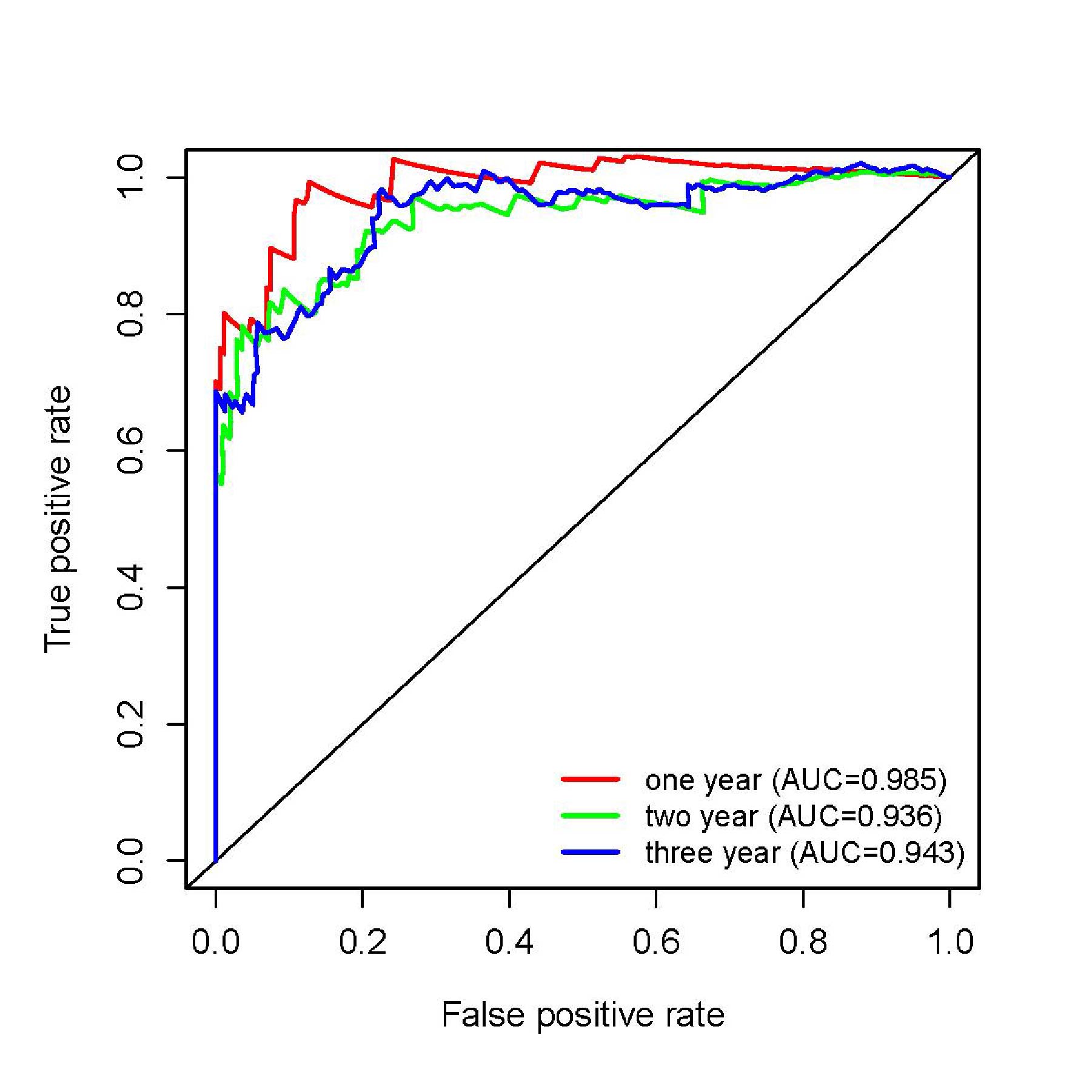

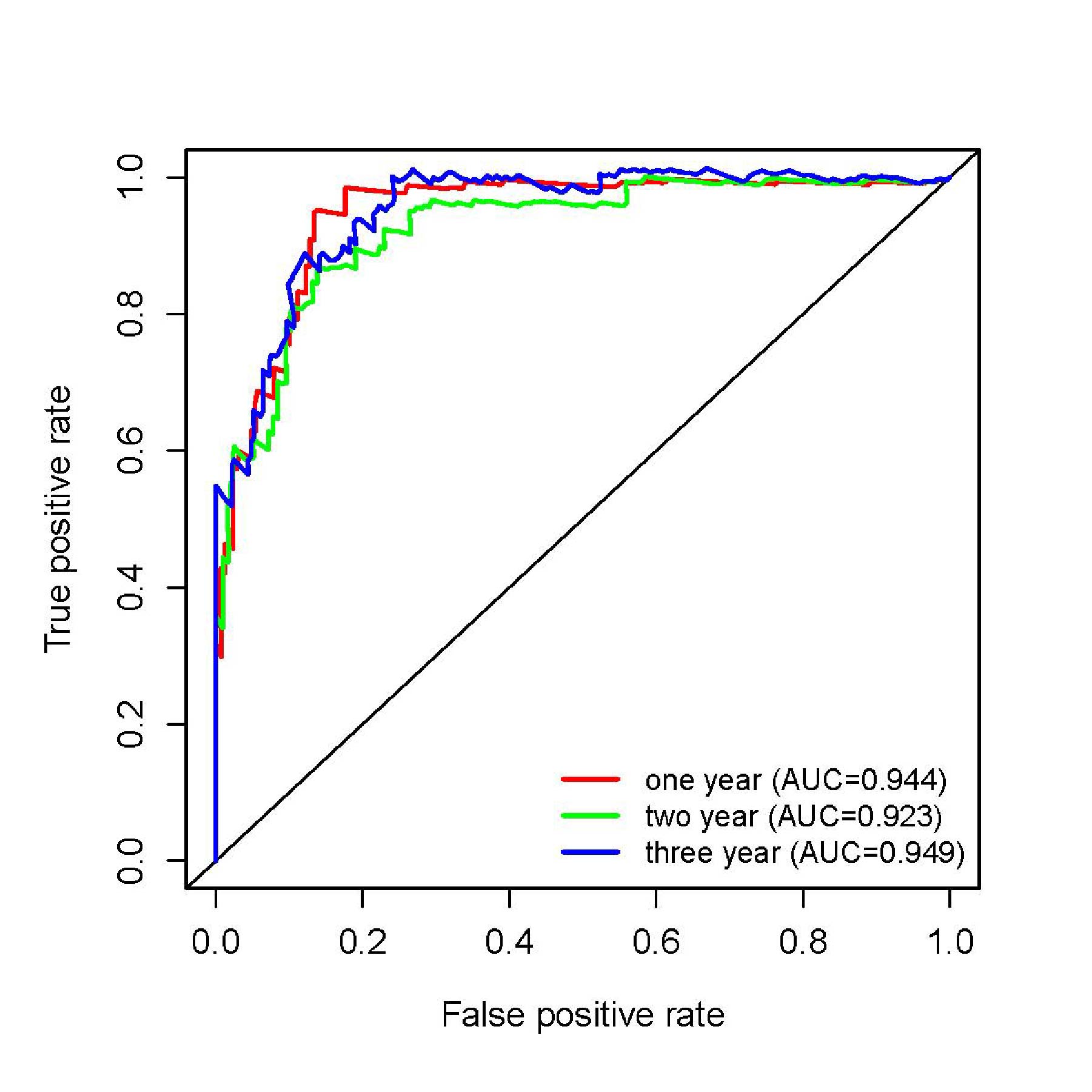


Figure S4A Figure S4B


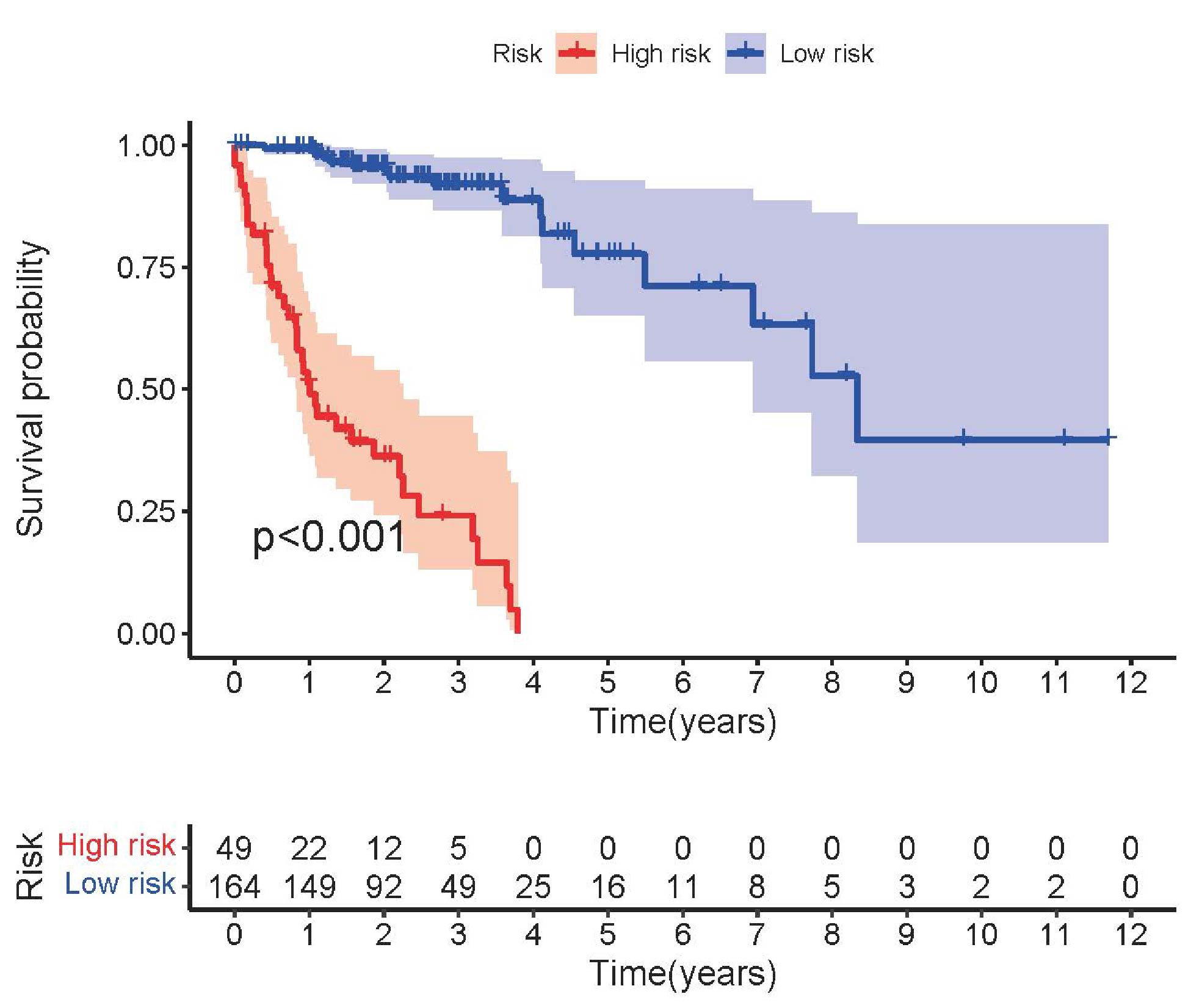

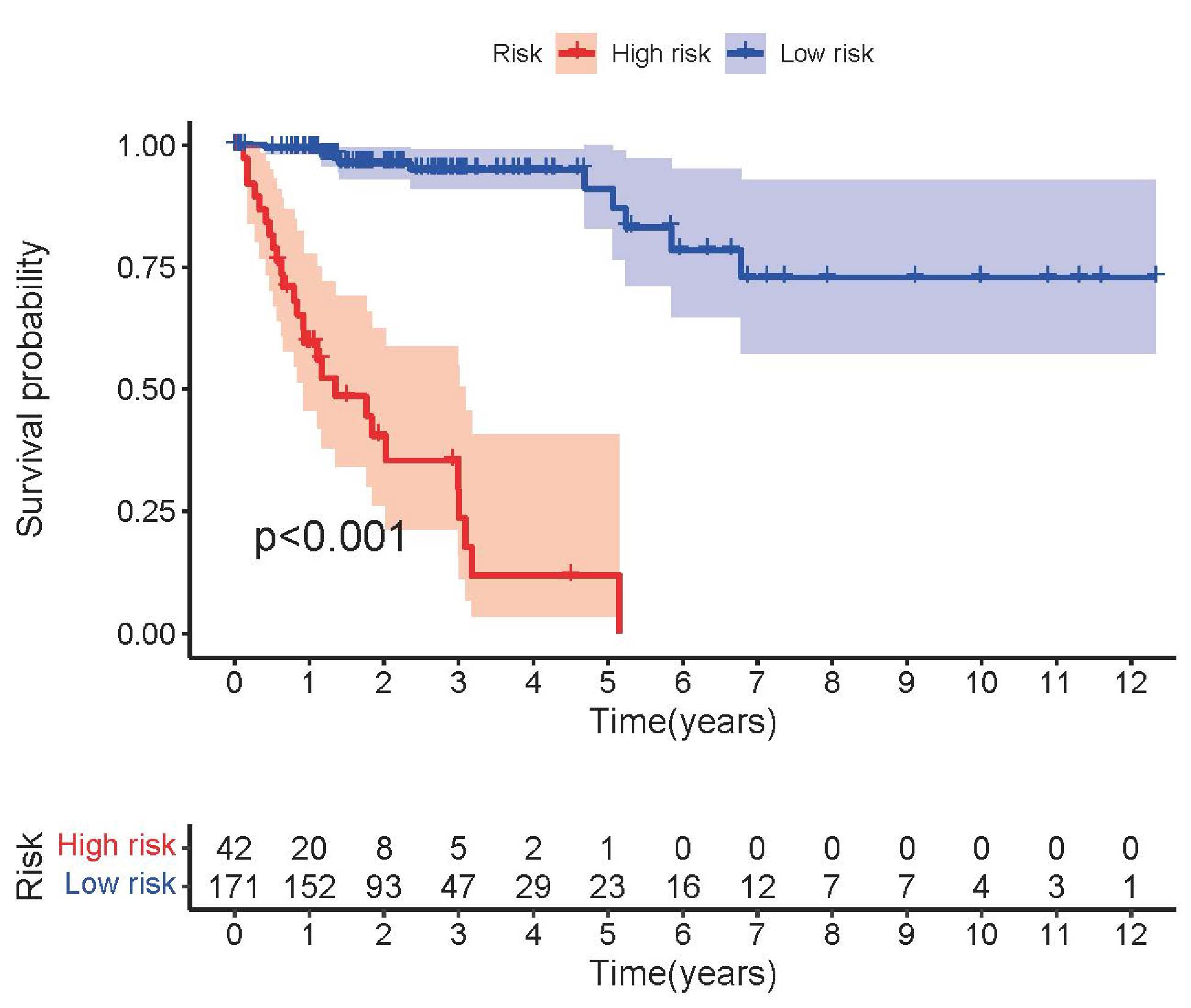


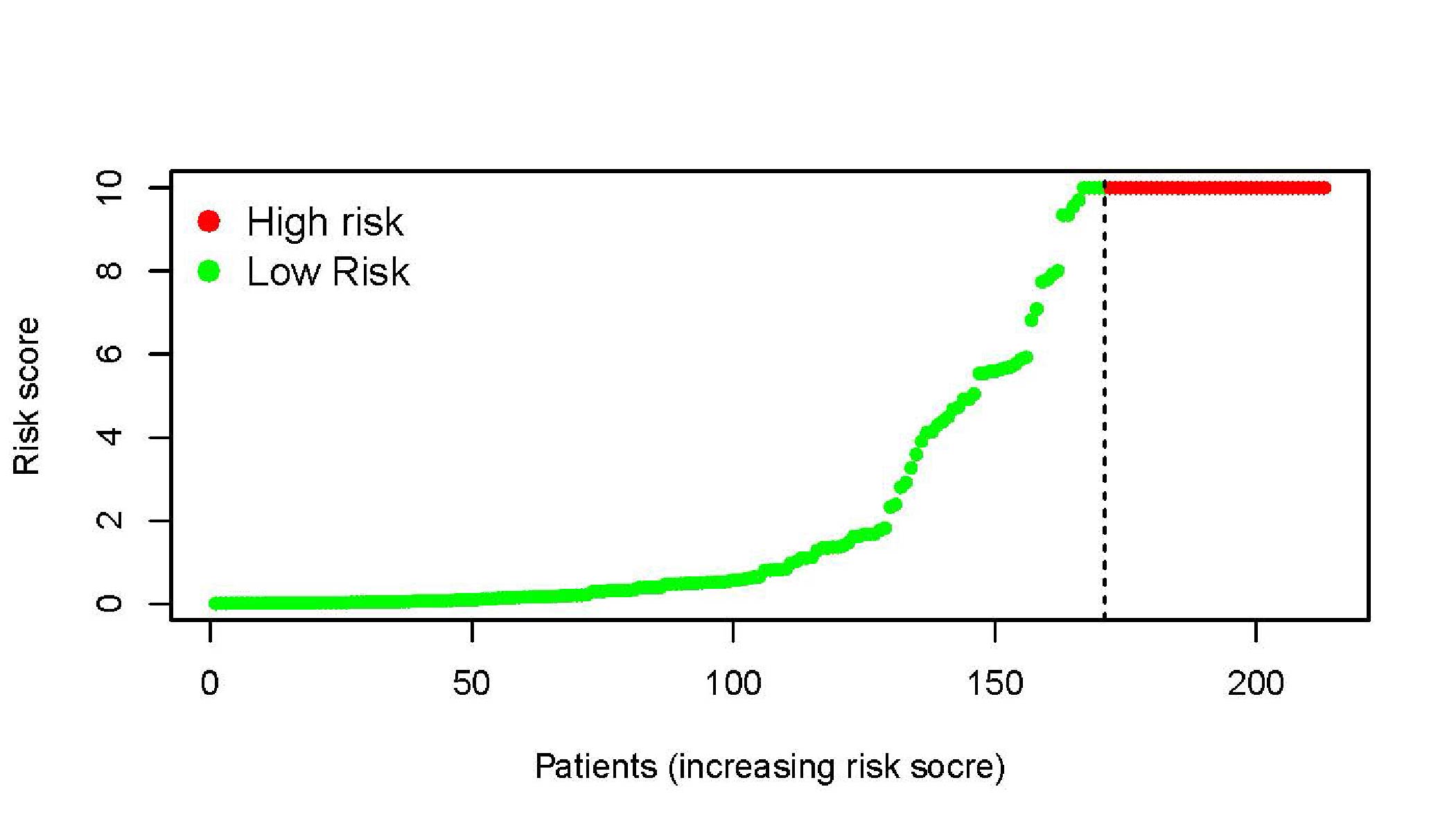

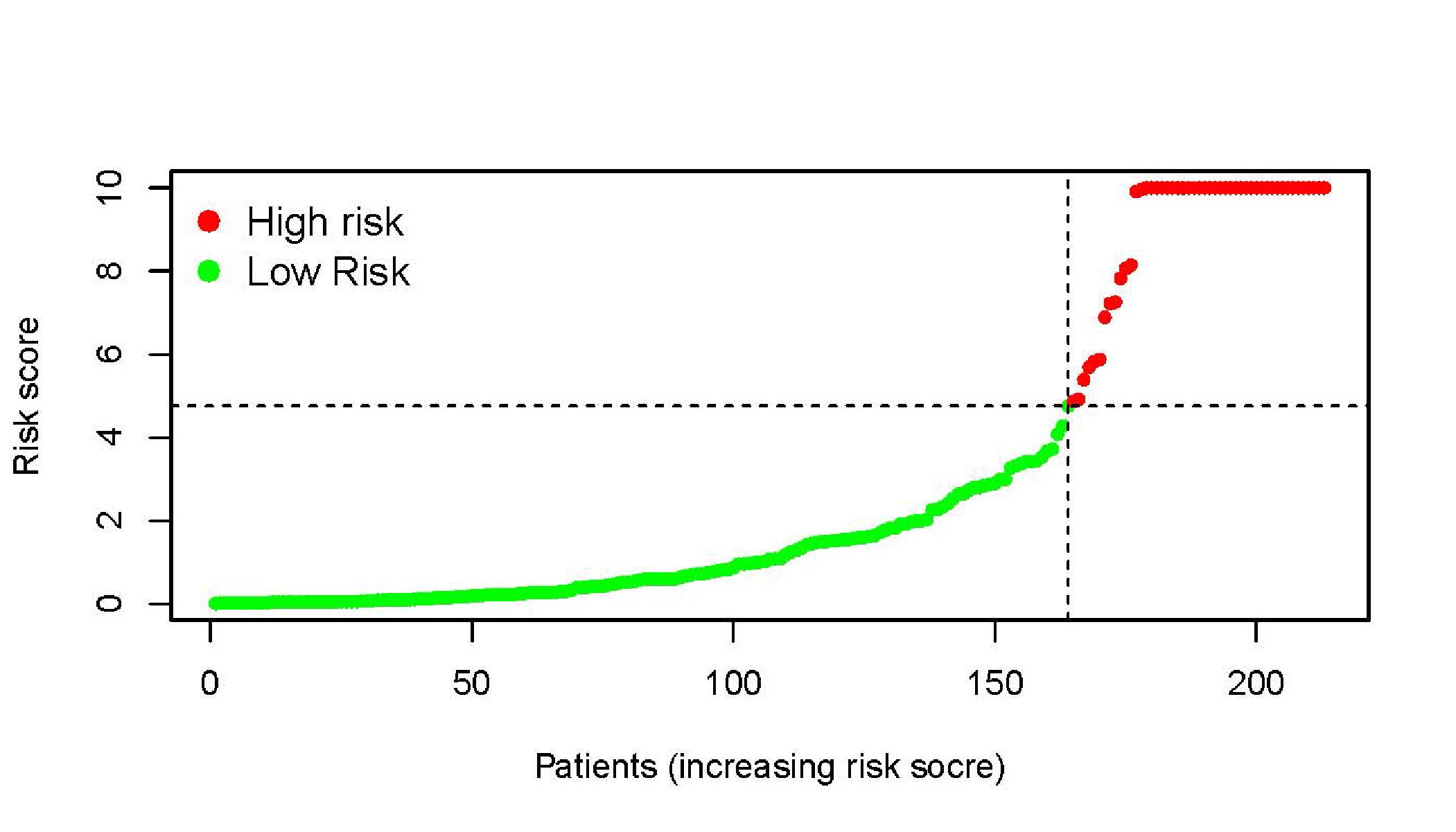
Figure S4C Figure S4D


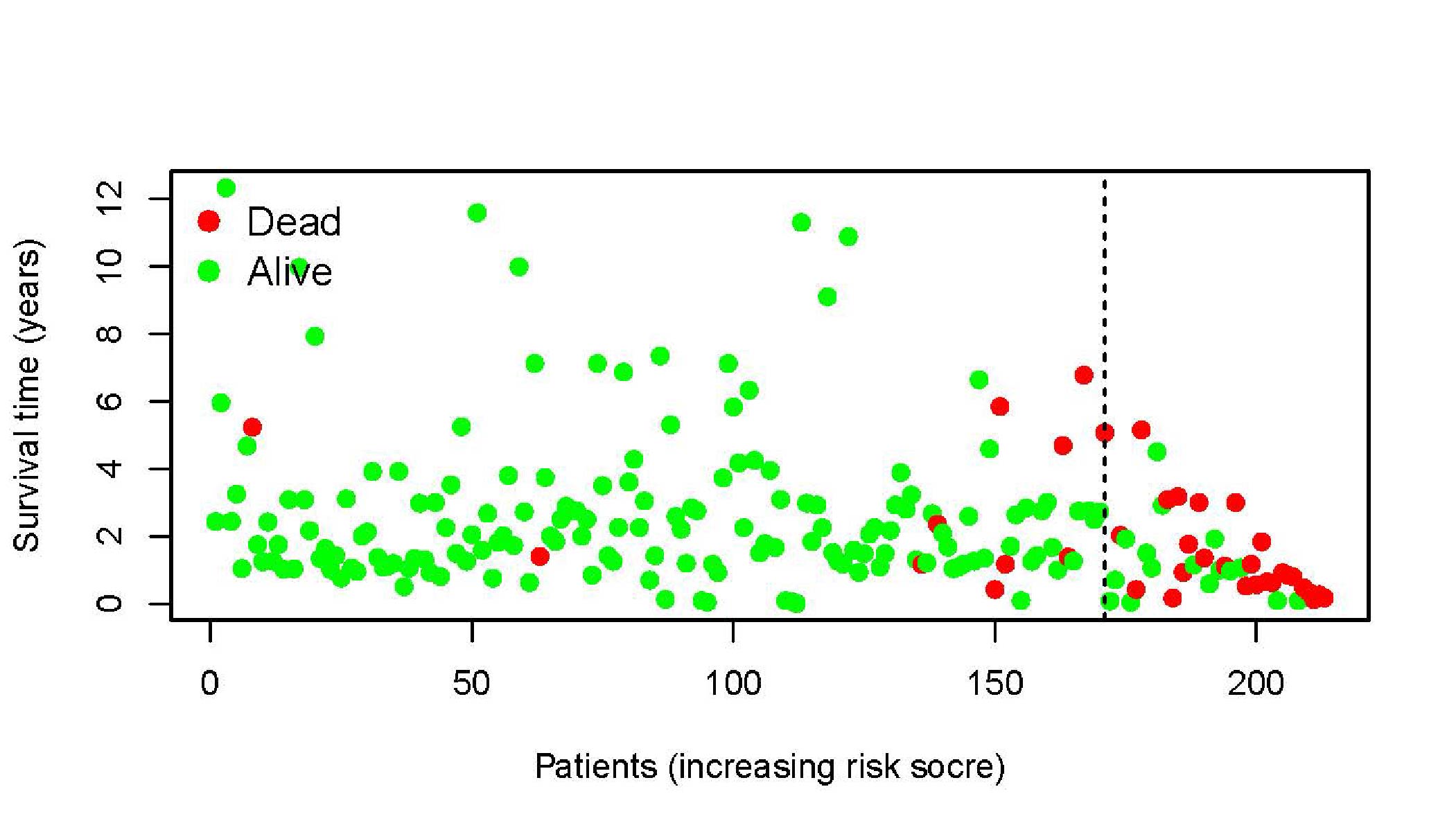

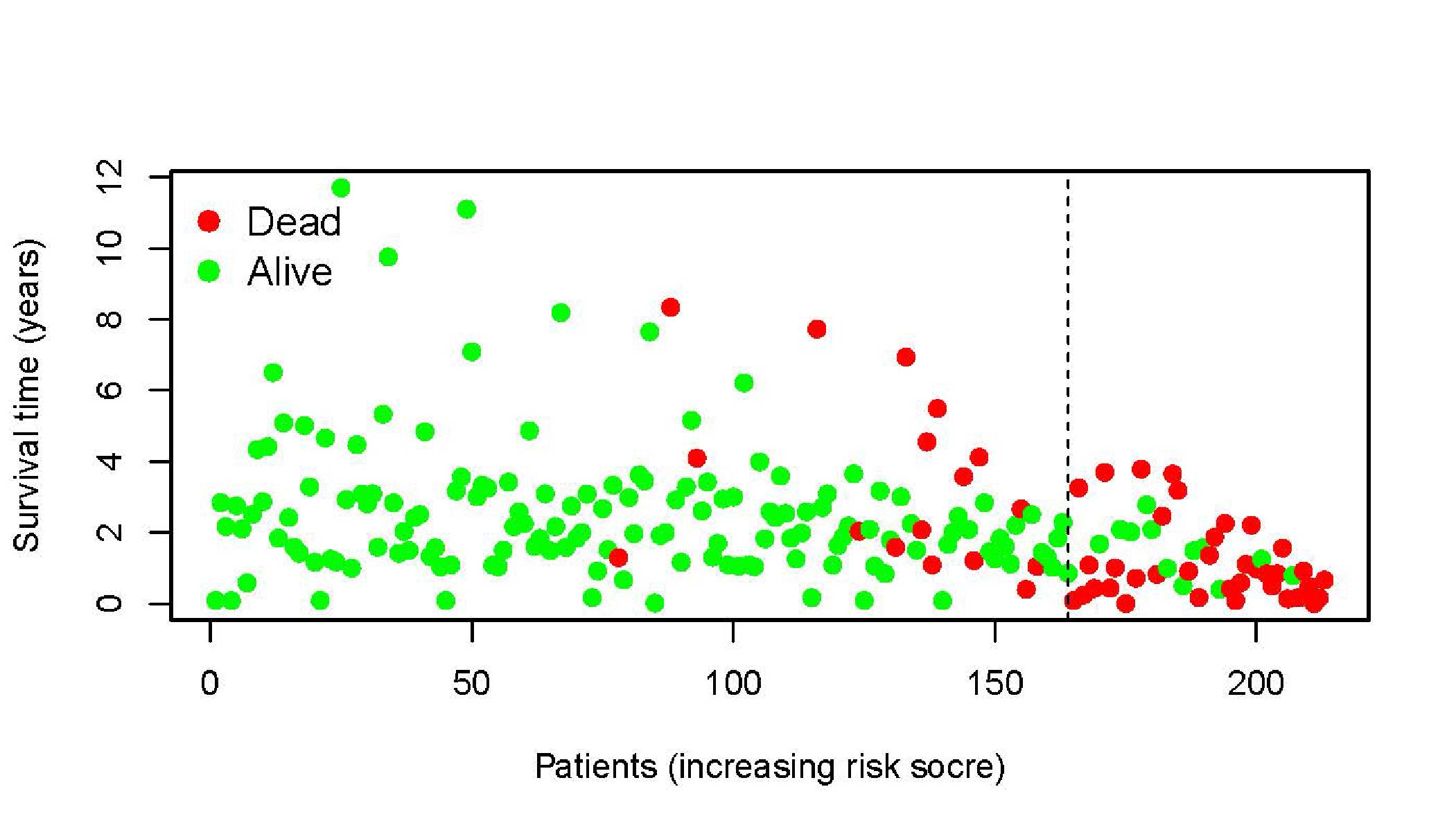
Figure S4E Figure S4F

**Additional file 2: Figures S2A-S2F, Figures S3A–S3F and Figure S4A-S4F**

Figure S2: (A) and (C) train queue lasso region, (B) and (D) test queue lasso region, (E), train queue forest map shows 14 DEirlncrna pairs determined by Cox proportional risk regression in the stepwise method, and (F) test queue forest map shows 13 DEirlncrna pairs determined by Cox proportional risk regression in the stepwise method

Figure S3: (A) Train queue Risk Score for 213 patients with COAD. the maximum inflection point is the cut-off point obtained by the AIC. (B), Test queue Risk Score for 213 patients with colon cancer. the maximum inflection point is the cut-off point obtained by the AIC. (C) The comparison of the 3-year ROC curve of the train cohort with other common clinical features shows the superiority of risk score. (D) The comparison of the 3-year ROC curve of the test cohort with other common clinical features shows the superiority of risk score. (E) Analysis of time-dependent receiver operating characteristic (ROC) curve in train queue. (F) Time dependent ROC curve analysis of test queue.

Figure S4: The prognostic model was validated in the train and test cohorts. (A) Overall survival (OS) of the train queue. (B) Total lifetime of test queue. (C) Risk score distribution in the train queue. (D) Risk score distribution in test queue. (E) Scatter diagram of survival status of train queue. (F) Scatter diagram of survival state of test queue.
